# High-Throughput Engineering of Nonribosomal Extension Modules

**DOI:** 10.1021/acschembio.3c00506

**Published:** 2023-11-20

**Authors:** Anna Camus, Maximilian Gantz, Donald Hilvert

**Affiliations:** Laboratory of Organic Chemistry, ETH Zurich, 8093 Zurich, Switzerland

## Abstract

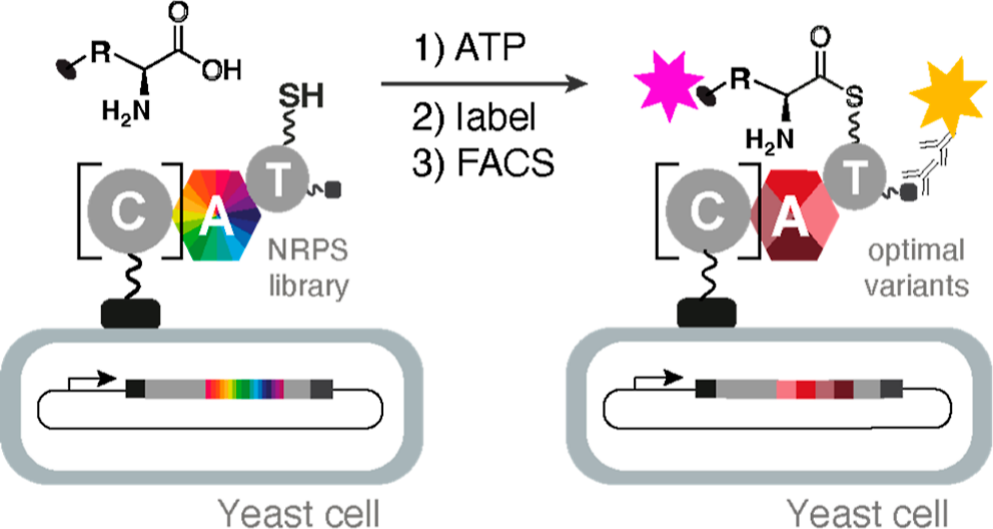

Nonribosomal peptides constitute an important class of natural products that display a wide range of bioactivities. They are biosynthesized by large assembly lines called nonribosomal peptide synthetases (NRPSs). Engineering NRPS modules represents an attractive strategy for generating customized synthetases for the production of peptide variants with improved properties. Here, we explored the yeast display of NRPS elongation and termination modules as a high-throughput screening platform for assaying adenylation domain activity and altering substrate specificity. Depending on the module, display of A–T bidomains or C–A–T tridomains, which also include an upstream condensation domain, proved to be most effective. Reprograming a tyrocidine synthetase elongation module to accept 4-propargyloxy-phenylalanine, a noncanonical amino acid that is not activated by the native protein, illustrates the utility of this approach for altering NRPS specificity at internal sites.

Nonribosomal peptides are biosynthesized in a thiotemplated fashion by large multimodular assembly lines called nonribosomal peptide synthetases (NRPSs).^1^ Individual modules contain dedicated domains that are responsible for substrate selection, activation, and elongation. For example, adenylation (A) domains choose the correct substrate, activate it as an aminoacyl adenylate, and transfer it to a thiolation (T) domain. Condensation (C) domains catalyze amide bond formation between the tethered building blocks, and once fully assembled, the natural product is usually released from the biosynthetic assembly line by a terminal thioesterase. Additional domains can further diversify the structure of the peptide by catalyzing epimerization, cyclization, and other reactions.^1−3^

The availability of detailed structural^4−9^ and mechanistic^2,8−10^ information on NRPS domains and modules has fueled efforts to engineer new synthetases for the sustainable production of novel peptides and therapeutic agents.^2,8,11^ We recently reported a high-throughput catalytic assay for NRPS A domains that enables rapid reprogramming of the substrate specificity of these gatekeeper enzymes.^12^ This assay involves functional display of a library of A–T bidomains on the surface of yeast cells and detection of successful amino acid activation and transfer to the phosphopantetheine (ppant) prosthetic group of the T domain by bio-orthogonal labeling of the yeast-bound product. Active variants are then separated from their inactive counterparts by fluorescence-activated cell sorting (FACS). Because up to 10^8^ variants can be screened in this way, large changes in substrate specificity can be achieved in a single experiment. Successful reprogramming of TycA, the initiation module of tyrocidine synthetase (Figure 1), for recognition of backbone-modified building blocks attests to the power of this approach.^12,13^

**Figure 1:**
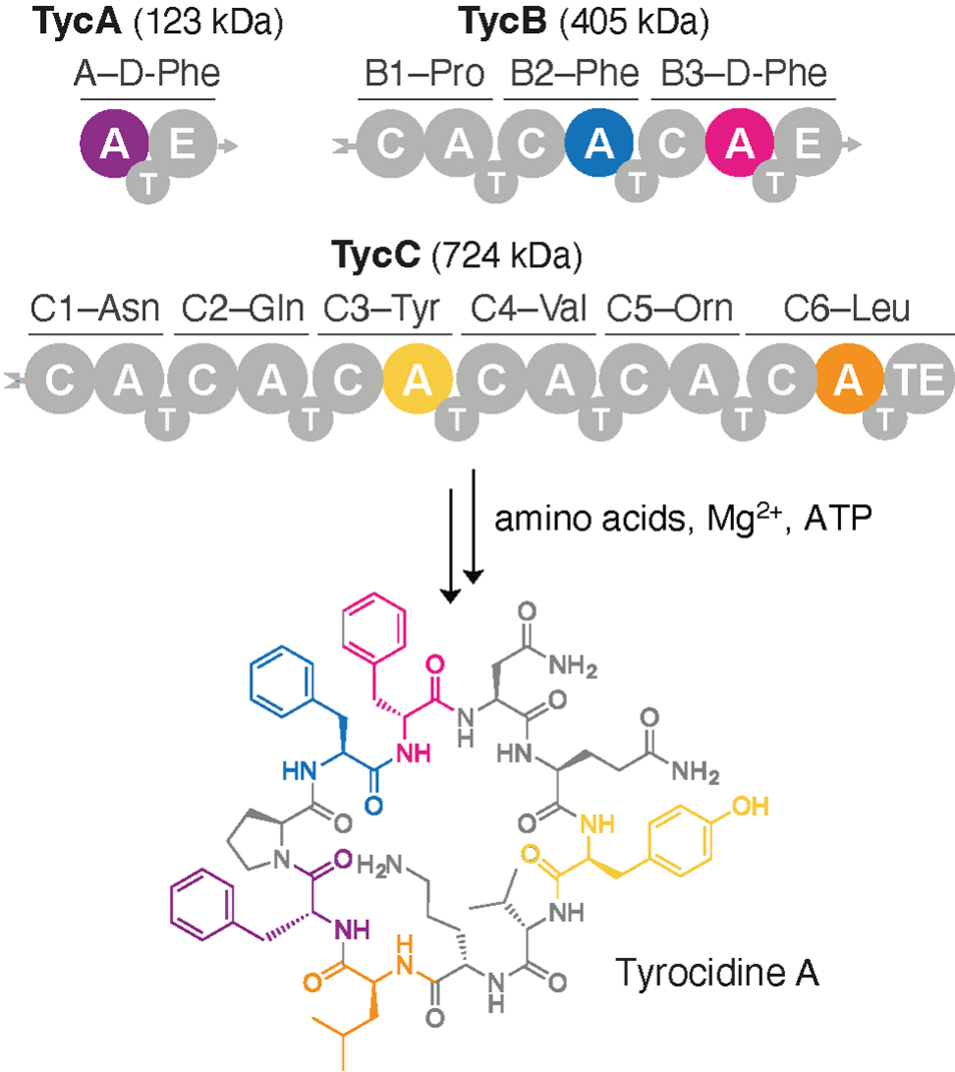
The antibiotic tyrocidine A is biosynthesized by a three-protein NRPS assembly line.^16^ Each protein consists of one or more modules composed of several functional domains, including condensation (C), adenylation (A), and thiolation (T) domains. Biosynthesis is initiated by TycA, and each subsequent module performs one round of peptide chain extension. Epimerase (E) domains in TycA and TycB3 invert the chirality of the phenylalanine residues at positions 1 and 4. Once assembly is complete, the terminal thioesterase (TE) in TycC6 releases the final cyclic product. Representative A–T and C–A–T constructs excised from TycA, TycB2, TycB3, TycC3, and TycC6 were successfully displayed on the surface of yeast cells.

To generalize this engineering tool for the modification of any site in a nonribosomal peptide, functional display of elongation and termination modules on yeast must be established. In contrast to standalone initiation modules like TycA, such modules are usually embedded in a synthetase, where they make interdomain contacts that may be important for activity. Consequently, extraction of the relevant enzymes requires a careful choice of excision sites to ensure proper functioning when the proteins are displayed on the surface of yeast.

Screening A domain libraries in high throughput also requires a noncanonical substrate that is equipped with a “clickable” handle that can be rapidly coupled to a fluorophore to provide a sensitive readout for A domain activity. For reprogramming of the Phe-specific TycA module, for example, a propargyloxy group was introduced into the side chain of phenylalanine (4-propargyloxy-l-phenylalanine, pPhe), which was accommodated by a single W227S mutation in the A domain.^14,15^ However, this tryptophan, which sits at the bottom of the Phe recognition pocket, is present only in some Phe-activating A domains (Figure S1). Thus, in the absence of promiscuous reactivity with pPhe or another clickable or fluorescent building block, only a limited set of A domains can be directly assayed by this approach, necessitating more extensive engineering.

Here we used alkyne-containing amino acids to show that A domains from one termination and three elongation modules from tyrocidine synthetase (Figure 1) can be displayed on yeast in functional form. High-throughput mutagenesis and screening of the Phe-specific A domain of the TycB2 module, which lacks the delimiting tryptophan in the substrate-binding pocket, enabled preferential activation of the noncanonical amino acid pPhe and its site-specific incorporation into the natural product with near wild-type efficiency. These results pave the way for efficient, on-demand production of diverse analogues of nonribosomal peptides by engineered assembly lines.

## Results

### Excision Strategy for Functional Yeast Display

For yeast display, we chose the TycB2, TycB3, TycC3, and TycC6 modules of tyrocidine synthetase, which incorporate l-Phe, d-Phe, l-Tyr, and l-Leu into the natural product (Figure 1). Since the functional display of an A domain with its cognate T domain on the surface of yeast is a minimal requirement for our assay,^12,13^ we focused initially on simple A–T constructs. If these could not be produced in functional form, we included the upstream C domain, which sometimes shares a large interface with its partner A domain,^17,18^ to stabilize the A–T bidomain. Previous efforts to engineer novel NRPS assembly lines by swapping A–T and C–A–T units^19−22^ guided these efforts.

For the A–T bidomain constructs, we made multiple sequence alignments of representative A domains and selected N-terminal excision sites within the upstream C–A linker region (Figure S2). SrfA-C, a full-length NRPS module that has been structurally characterized (PDB: 2VSQ),^18^ served as a reference. To provide the A domain with the conformational flexibility required for successful adenylation and thioesterification,^23−25^ we chose a site immediately following the only α helix in the C–A linker (Figure S2a,c). Due to the reported decrease in A domain activity when cut sites further upstream of this helix are used,^26^ larger fractions of the linker region were not included. Based on the crystal structure of the T–C bidomain of TycC5 and TycC6 (PDB: 2JGP),^27^ a C-terminal excision site directly after a conserved aliphatic residue (e.g., Leu5201 in TycC6) was chosen (Figure S2b,d).

For C–A–T constructs, the N-terminal excision site was selected immediately after the upstream T domain and including the entire linker segment and C domain as described by Mootz et al.^28^ The same C-terminal excision site that was used for the successful display of the TycA A–T bidomain was adopted since the T domain in that construct was properly folded and active in the absence of its associated E domain.^12,13^

### Functional Display of NRPS Modules on Yeast

**Figure 2:**
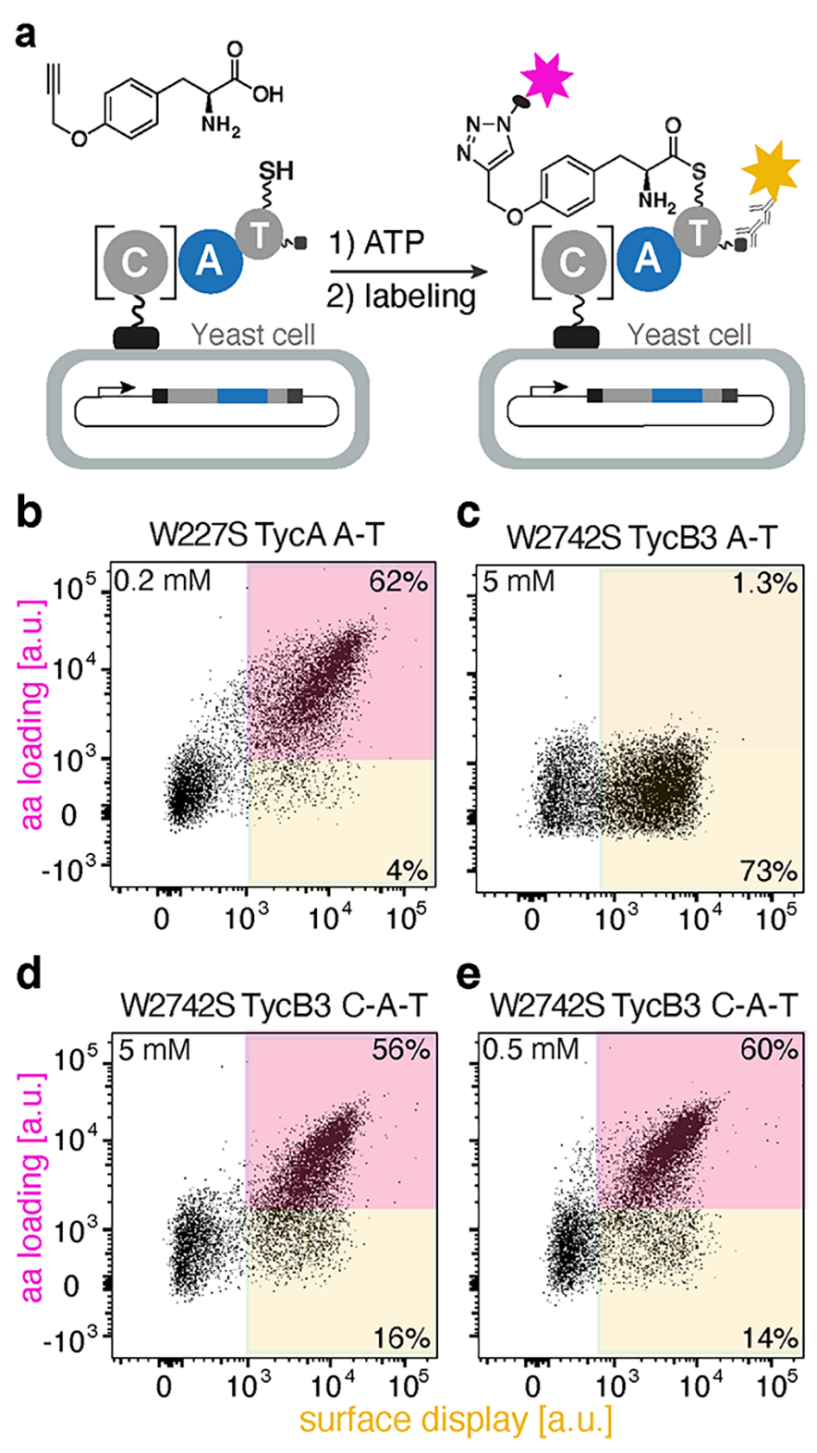
TycB3 A domain activity on yeast cells. (a) Yeast surface display of an A–T bidomain or a C–A–T tridomain, followed by substrate loading and bio-orthogonal labeling. Flow cytometric analyses of (b) the W227S TycA A–T bidomain with 0.2 mM pPhe; (c) the W2742S TycB3 A–T bidomain with 5 mM pPhe; and (d) the W2742S TycB3 C–A–T tridomain with either 5 mM pPhe or (e) 0.5 mM pPhe.

We started with the third module of the TycB protein (Figure 1), TycB3, a homologue of TycA with 61% sequence identity. Like TycA, its A domain has a conserved tryptophan at the bottom of the phenylalanine-binding pocket, and replacement of this residue with serine in tyrocidine synthetase similarly enables efficient loading of the clickable amino acid pPhe in vitro.^15^ For yeast display, we excised the A domain together with its cognate T domain and introduced the permissive Trp-to-Ser mutation at position 2742. The resulting A–T bidomain, fused to the C terminus of the yeast mating factor Aga2p, was cloned into a galactose inducible vector and transformed into EBY100 yeast cells.^29^ After producing the surface-displayed protein (Figure 2a), cells were first incubated with coenzyme A (CoASH) and the recombinantly produced 4′-phosphopantetheinyl transferase Sfp to install the phosphopantetheine cofactor, and then with 5 mM pPhe and 0.1 mM ATP for 10 min as described for the analogous W227S TycA system (Figure 2b).^12,13^ Following bio-orthogonal biotinylation of the sample,^30,31^ streptavidin derivatized with an R-phycoerythrin dye (R-PE) was added to visualize any labeled yeast. Despite efficient protein display, covalent attachment of pPhe to the cells was not detected by flow cytometry (Figure 2c).

The lack of activity for the displayed A–T bidomain was unexpected given efficient in vitro biosynthesis of the corresponding pPhe-containing tyrocidine analogue with W2742S TycB.^15^ We therefore prepared a second construct that included the upstream C domain to mitigate potential instability (Figures 2a and S3). Even though the resulting W2742S TycB3 C–A–T module is considerably larger than the original A–T construct (∼130 vs ∼80 kDa), it was efficiently displayed on EBY100 yeast cells. Moreover, when the cells were assayed under the same conditions as before, high activity with pPhe was observed by flow cytometry, with robust surface labeling at both 5 and 0.5 mM pPhe (Figure 2d,e).

**Figure 3:**
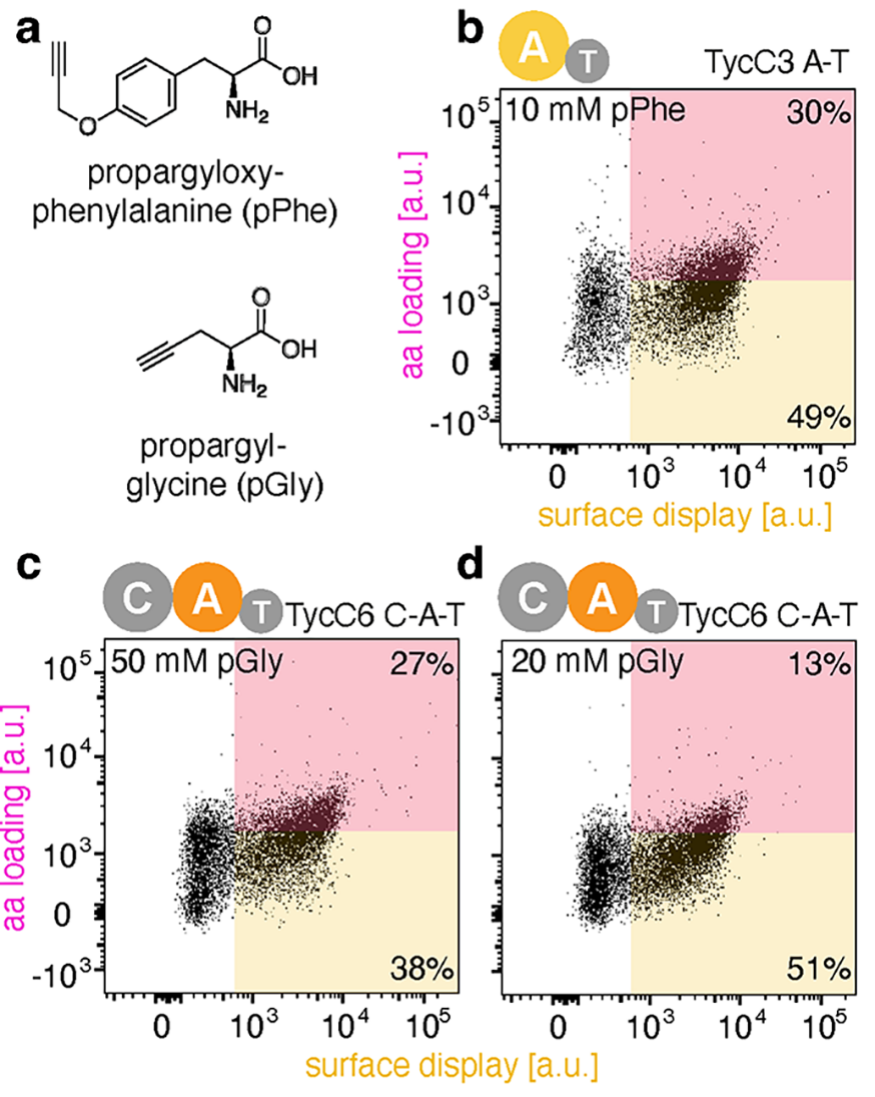
Promiscuous activity of the TycC3 elongation and TycC6 termination modules with “clickable” amino acids. (a) Chemical structures of pPhe and pGly. (b) Dot plot of the TycC3 A–T bidomain with 10 mM pPhe. (c) Dot plots of the TycC6 C–A–T tridomain with 50 mM pGly and (d) 20 mM pGly. Black dots in the pink box illustrate the fraction of the A domains displayed on the yeast cell surface that accept pPhe or pGly as a substrate.

In contrast to TycB3, the A–T bidomain from TycC3, which normally activates tyrosine and shares only 45% sequence identity with the A domain of TycA, could be displayed on yeast in functional form in the absence of its partner C domain. This construct showed promiscuous activity when incubated with 10 mM pPhe (Figure 3a,b). The observed activity is lower than that of the optimized W2742S TycB3 C–A–T construct, likely because the bulky propargyl group of pPhe is not optimally accommodated in the Tyr recognition pocket. Nonetheless, this activity should be sufficient for engineering improved variants capable of activating a variety of para-substituted phenylalanine analogues^14,15^ and substrates with altered backbones.^12,13^

Like TycB3, the termination module of the synthetase, TycC6 (46% sequence identity with the A domain of TycA), was successfully displayed as a C–A–T construct. Its A domain activates l-Leu, which is considerably smaller than the aromatic amino acids recognized by the other A domains tested. We therefore used the clickable aliphatic amino acid propargyl glycine (pGly) instead of pPhe to screen for catalytic activity (Figure 3a). When cells displaying the TycC6 module were incubated with 50 and 20 mM pGly, the displayed module was found to activate pGly in a concentration-dependent manner, albeit with modest efficiency (Figure 3c,d). As for TycC2, this promiscuous activity represents an attractive starting point for future rounds of mutagenesis and high-throughput screening.

### Reprograming the Specificity of an Elongation Module

If an A domain does not naturally accept a clickable amino acid as a substrate, then the yeast display assay can be used to provide this capability. This was the case for the A domain of the second TycB module, TycB2, which is only distantly related to the Phe-specific A domains of TycA (45% sequence identity). The native enzyme does not recognize pPhe and lacks the delimiting tryptophan at the bottom of the Phe-binding pocket. Multiple sequence alignment of all ten A domains from tyrocidine synthetase (Figure S3a), plus a larger selection of A domains specific for aromatic amino acids^32^ (Figure S3b), revealed that only ∼30% have a tryptophan at position 227 (TycA numbering) that could be used as a target for simple substitution. The rest exhibit high variability at this site.

**Figure 4:**
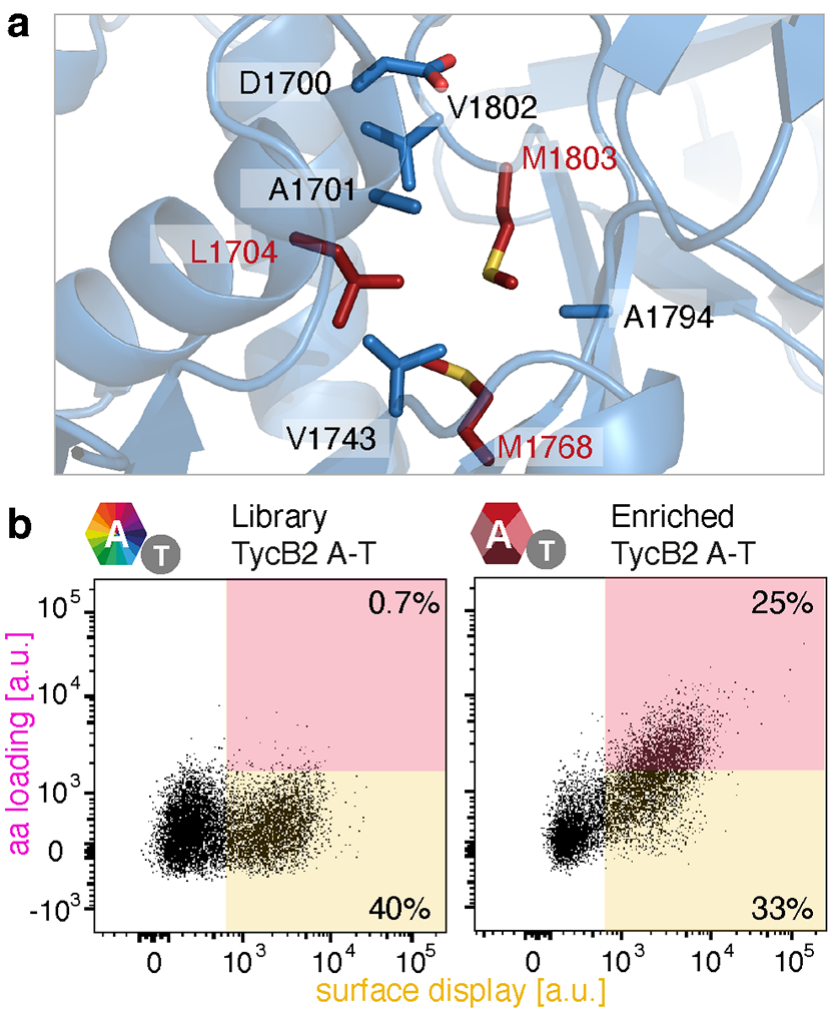
Mutagenesis and screening strategy for altering A domain specificity. (a) Schematic representation of the binding pocket of the TycB2 A domain generated by SWISS-Model using LgrA (PDB: 5ES6)^23^ as a template. Residues lining the binding pocket are shown as sticks. The three residues targeted for substitution are highlighted in red. (b) Dot plots of the starting (left) and enriched (right) library incubated with 5 mM pPhe.

To engineer TycB2 to accept pPhe as a substrate, we excised its A–T bidomain for display on yeast as described for TycC3 (Figure S3). Since a crystal structure of this A domain was not available, we generated a protein homology model to guide library design (Figure 4a). In the model, Leu1704 corresponds to Trp227 in TycA. This residue and two nearby methionines at positions 1768 and 1803 were subjected to saturation mutagenesis using NNK codons to increase space for the propargyloxy moiety of pPhe. The resulting library, which contained approximately 33,000 gene variants, was used to transform EBY100 yeast cells. After the corresponding proteins were produced and displayed on the cell surface, the cells were incubated with CoASH and Sfp, followed by the addition of ATP and pPhe. Cells producing active variants were biotinylated and enriched by FACS (Figure 4b, pink box). Two sorting rounds were performed under increasingly stringent conditions, decreasing the substrate concentration in the second sort from 5 to 0.5 mM and adding 0.5 mM l-Phe as a competitor.

Sequencing of representative variants from the enriched population revealed that mutation of Leu1704 was not necessary to accommodate the propargyloxy group of pPhe (Figure S4). Instead, both methionine residues were replaced by smaller amino acids. For example, Met1768 was mutated to Ala, Gly, or Cys, whereas Met1803 was replaced with Ile or Val. To identify the best variant for in vitro characterization, relative activities were assessed by performing the yeast display assay with each candidate separately using 0.5 mM pPhe in the presence of competing 0.5 mM l-Phe. Flow cytometry showed that the variant with the M1768A and M1803I mutations was the most active and selective (Figure S5).

**Figure 5:**
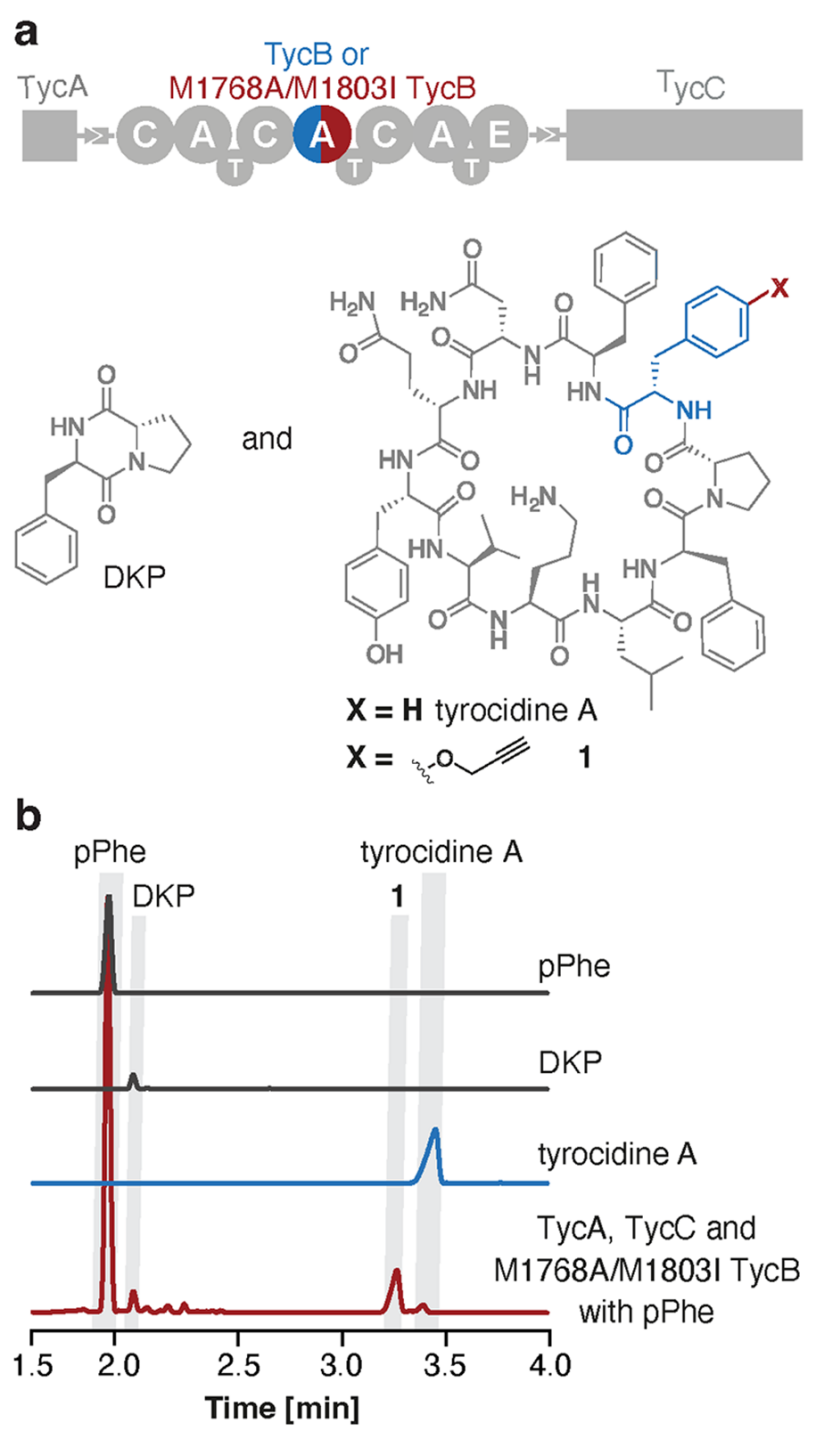
Biosynthesis of a “clickable” tyrocidine derivative. (a) Schematic representation of tyrocidine synthetase which produces DKP and the cyclic decapeptide. (b) HPLC chromatograms of pPhe (gray), DKP (gray), and tyrocidine A (blue) standards and the product (red) of the biosynthetic reaction performed with TycA, M1768A/M1803I TycB, and TycC in the presence of pPhe.

The M1768A and M1803I mutations were cloned into the full-length TycB protein to enable biosynthesis of a propargylated tyrocidine analogue (Figure 5). All three proteins of the synthetase were heterologously produced in *Escherichia coli*, purified, and posttranslationally modified as previously described (Figure S6).^15^ Biosynthetic reactions were performed by incubating TycA, M1768A/M1803I TycB, and TycC with ATP, Mg^2+^ and all component amino acids, including pPhe. To increase product yield,^15^ inorganic pyrophosphatase, which hydrolyzes pyrophosphate, a side product of amino acid adenylation, to inorganic phosphate, was added to shift the equilibrium toward the full-length natural product analogue.

HPLC analysis of the biosynthetic reaction mixture revealed the formation of a new compound as the major product (Figure 5b). LC–MS/MS experiments allowed definite assignment of the corresponding peak to the novel tyrocidine analogue **1**, in which the l-Phe at position 3 of the sequence was replaced site-specifically by pPhe (Figure S7). As is typical for such reactions,^13,15^ small amounts of DKP and traces of native tyrocidine A were observed as minor side products (Figure 5b).

## Conclusions and Perspectives

Robust methods for modifying nonribosomal assembly lines could provide biosynthetic access to novel peptide analogues with improved properties or altered bioactivities. To that end, diverse strategies for engineering NRPSs have been developed, including module insertion, module deletion, module exchange, and subdomain swaps.^19−21,33−38^ As a less invasive alternative, the substrate specificity of individual A domains can also be altered through mutagenesis and screening.^12−14,39−42^ Because of its high throughput, the FACS-based approach that we recently introduced to monitor the catalytic activity of A domains^43^ could greatly increase the utility of the latter approach.

To monitor both reactions catalyzed by A domains, namely, substrate adenylation and thioesterification, the A domain of interest must be displayed on the surface of yeast cells together with its cognate T domain. This arrangement physically links genotype and phenotype, because only functional variants are able to activate the substrate and covalently tether it to the host cell. Following bio-orthogonal labeling, many millions of variants can be screened in a single experiment by FACS, allowing efficient exploration of sequence space. The current study demonstrates that this approach is not restricted to the previously studied TycA initiation module^12,13^ but can also be applied to A domains from elongation and termination modules.

Our results show that both A–T and C–A–T modules from tyrocidine synthetase can be functionally displayed on yeast. We anticipate that the benefits of this eukaryotic expression system will also extend to modules of many other bacterial and fungal synthetases. That said, problems with folding or stability of the display constructs can arise as suggested by the observation that some A–T bidomains worked well (TycA, TycB2, and TycC3), whereas others did not (TycB3). Although it might be possible to rescue inactive A–T bidomains by choosing more appropriate excision sites^44^ or exploiting the FACS assay for directed evolution,^45,46^ simply including the cognate upstream C domain in the display construct provided an easy fix in the case of TycB3. Despite being ∼50 kDa larger in size than the A–T bidomains, C–A–T modules exhibited excellent display efficiency, likely because of the stabilizing effects associated with the C–A domain interface, which can be quite extensive (up to ∼1600 Å^2^).^17,18^ Because C–A–T modules also preserve catalytically relevant conformational changes and other interactions needed for proper communication between adjacent domains, they may prove to be the excision units of choice for many applications.

For most of the A domains we tested, promiscuous activity with a propargylated substrate could be observed either directly or after introducing permissive active site mutations. As previously described for TycA,^12,13^ such activity can be exploited to re-engineer these modules for recognition of side chain and backbone-modified building blocks. Even if detectable starting activity is absent, as was the case for TycB2, the FACS-based assay can be used to install it. Although screening a relatively small three-residue library sufficed to reprogram TycB2 for the recognition of pPhe, exhaustive screening of much larger libraries would be feasible for more demanding engineering challenges. Notably, the best pPhe-specific TycB2 variant functioned effectively in the context of the entire synthetase, enabling the efficient incorporation of this clickable amino acid into the natural product. Such handles have become indispensable tools in synthetic biology,^47−49^ and the ability to place them site-specifically within a peptide natural product could be broadly useful for isolation, imaging, and mechanistic applications.

It is also worth noting that successful yeast display of entire NRPS modules creates opportunities beyond monitoring amino acid loading. For example, condensation reactions can also be assayed in high throughput. We recently showed that a displayed C–A–T domain equipped with a short docking domain could productively interact with an upstream module in solution to produce amide products tethered to the yeast surface, an activity we exploited to alter the substrate specificity of the displayed C domain.^50^ Extension to other bio-orthogonal labeling methods to enable variation of substrate side chain preferences or harnessing the power of iterative directive evolution^45,46^ would further expand the range of problems that can be tackled with this assay platform.

The design principles and tools developed in this study lay the foundation for a scalable, sustainable, and minimally invasive method for engineering NRPS machinery for modifications at any position in peptide natural products. In addition to providing a deeper understanding of assembly line biosynthesis, a frontier area in enzymology, this technology promises to foster the discovery and production of life-saving therapeutic agents.

## Materials and Methods

Detailed procedures for molecular cloning, protein production/purification, and MS/MS analysis can be found in the Supporting Information.

### Homologous Recombination in Yeast

Competent EBY100^29^ cells were prepared using the Frozen-EZ Yeast Transformation II Kit (Zymo Research). The competent cells were combined with the pCTRB vector backbone, predigested with NdeI and XhoI (around 100 ng), and the relevant PCR product in a 1:6 ratio. Transformation was performed according to the supplier’s protocol. The cell suspension (100 μL) was plated onto SD-CAA agar plates and incubated for 2 days at 30 °C.

### Plasmid Sequencing

Assembled plasmids and plasmids from libraries or sorts were isolated using a ZR-Plasmid Miniprep Classic Kit (Zymo Research). To lyse yeast cells efficiently, cell pellets were suspended in the Zymo P1 buffer to which 100 μg of glass beads were added (diameter 0.5 mm, Carl Roth GmbH + Co). The neutralization steps and plasmid purification were performed as recommended by the manufacturer. Plasmids were subsequently amplified by transformation of electrocompetent XL1 blue cells, reisolated using the ZymoPURE Plasmid Miniprep Kit, and sequenced by Microsynth AG (Switzerland). Glycerol stocks (20% w/v) of EBY100 and the XL1 blue strains transformed with the plasmid were prepared and stored at −80 °C.

### Transformation of Yeast with Libraries

Plasmid libraries were generated by the same homologous recombination approach that was used to prepare display plasmids for individual variants. However, to increase transformation efficiency, 400 μL of freshly prepared electrocompetent EBY100 yeast cells^29^ were treated with 1 μg pCTRB backbone, predigested with NdeI and XhoI, and 2 μg of the library PCR product as described by Benatuil et al.^51^ Electroporation was conducted in 2 mm cuvettes (2.5 kV and 25 μF), immediately followed by addition of 8 mL of yeast extract-peptone-dextrose (YPD)/1 M sorbitol (1:1 ratio) and incubation of the resulting suspension at 30 °C and 230 rpm for 1 h. After centrifuging the cells at 3000*g* for 3 min, the pellet was suspended in 250 mL of SD-CAA medium containing phosphate buffer (100 mM, pH 6), glucose (Fluorochem, 20 g/L), Difco yeast nitrogen base without amino acids (BD, 6.7 g/L), and casamino acids (BD, 5 g/L). A dilution series was prepared, and libraries diluted 10^3^- and 10^4^-fold were plated on SD-CAA agar plates. The transformation efficiency was determined after incubation for 2 days at 30 °C. The plasmids for single clones from this initial library were isolated and sequenced as described above to verify library quality. The remaining 250 mL of the library was incubated at 30 °C and 230 rpm for 20–24 h until an OD_600_ of ∼2 was reached. Approximately 1 mL of this library was diluted in 25 mL of SD-CAA medium to obtain an OD_600_ of ∼0.1. The library was stored in 100 mL Erlenmeyer flasks at 4 °C prior to display and screening. A 20% (w/v) glycerol stock of the EBY100 culture containing the respective library was prepared and stored at −80 °C.

### Display of A–T Bidomains and C–A–T Tridomains on Yeast

To display NRPS constructs on the surface of yeast cells, the protocols of Boder and Wittrup^29^ and Niquille et al.^12^ were followed. In brief, a single EBY100 colony transformed with the plasmid of interest was inoculated in 3 mL of SD-CAA medium and incubated at 30 °C for 16 h. The overnight culture typically exhibited an OD_600_ value between 4 and 8. A 100–200 μL aliquot of this culture was added to 3 mL of SD-CAA, and the resulting sample, which had an initial OD_600_ of ∼0.2, was incubated at 30 °C and 230 rpm for 4–5 h until an OD_600_ of ∼1 was obtained. Protein expression was induced by exchanging the SD-CAA medium with 3 mL of SG-CAA containing phosphate buffer (100 mM, pH 6), galactose (AppliChem, 20 g/L), Difco yeast nitrogen base without amino acids (BD, 6.7 g/L), and casamino acids (BD, 5 g/L). Protein expression was performed at 16 °C and 230 rpm for around 20 h. For libraries, the stored 25 mL culture was incubated directly at 30 °C for 4–5 h and the pellet was resuspended in 25 mL of SG-CAA instead of 3 mL.

### Yeast Surface Display Assay

To assay A domain activity of NRPS modules displayed on yeast, a protocol adapted from Niquille et al.^12^ was used with optimized reagent amounts and reaction conditions. Induced cell suspensions (200 μL) harboring the variant of interest were pelleted and washed twice. The cell pellet was then incubated in a solution (50 μL) of coenzyme A (BioChemica, 0.2 mM) and the Sfp 4′-phosphopantetheinyl transferase^52,53^ (15 μL) in pH 7.4 PMB buffer [40 mM Na_2_HPO_4_, 7.2 mM NaH_2_PO_4_·1H_2_O, 137 mM NaCl, 2.7 mM KCl, and 1 mM MgCl_2_ and bovine serum albumin (1 mg mL^–1^)] for 5 min at 25 °C. Before adding the amino acid substrate, the cells were pelleted again, washed with PMB buffer, and centrifuged at 3000*g* and 4 °C for 30 s, and the supernatant was discarded. Reaction with the amino acid substrate was initiated by adding variable amounts of either 4-propargyloxy-phenylalanine (pPhe) or propargyl-glycine (pGly) to the cell pellet resuspended in 200 μL of PMB buffer containing ATP (0.1 mM, Meiya pharmaceuticals). After incubation for 5 min at 25 °C, the reaction was stopped by centrifugation and removal of the supernatant, followed by washing once with PMB (180 μL). Cells covalently presenting the substrate on the displayed T domain were then bio-orthogonally labeled by incubation with an azide-PEG3-biotin conjugate (Sigma, 20 μM), CuSO_4_ (25 μM), bathophenanthrolinedisulfonic acid (50 μM), and freshly prepared l-ascorbic acid (0.5 mM) in PMB (50 μL) at 4 °C for 2 h. Labeling was stopped by centrifugation and removal of the supernatant, followed by washing twice with PMB (180 μL). The cells were pelleted by centrifugation, resuspended in PMB (20 μL), and incubated with monoclonal mouse anti-*c*-myc antibody 9E10 (3.2 μg/mL, Roche) for 10–20 min on ice. The cells were then pelleted by centrifugation, resuspended in PMB (20 μL), and labeled with a goat antimouse IgG-FITC antibody (8.8 μg/mL, Sigma-Aldrich) and a streptavidin-R-phycoerythrin conjugate (20 μg/mL, Life Technology) at 4 °C for another 10 min. The cells were pelleted, washed twice, dissolved in 500–800 μL PMB buffer, and transferred to a round-bottom tube with a cell strainer cap (Falcon, 5 mL). They were kept on ice until analyzed by flow cytometry on an LSRFortessa (BD) Cell Analyzer.

For sorting experiments, 1–2 mL of the SG-CAA cell suspension was used, and the volumes in the assay steps were scaled up accordingly. The assay for the TycB2 AT library was performed with 5 mM pPhe in the first sort, followed by 0.5 mM pPhe and 0.5 mM l-Phe, which acts as a competitor, in the second sort. The cells were resuspended as before in a PMB buffer. Cells displaying active variants were separated from inactive variants based on the FITC and PE fluorescent signals using an FACSAria III (BD) Cell Sorter. The sorting gates were always adjusted according to the populations of unlabeled cells and cells labeled with (R)-PE or FITC alone. The sorted cells were collected in 15 mL Falcon tubes filled with 8 mL of SD-CAA medium and transferred into sterile 100 mL Erlenmeyer flasks. The falcon tubes were washed twice with 3 mL of SD-CAA. The Erlenmeyer flasks were incubated at 30 °C and 230 rpm for 24–48 h until an OD_600_ of 2–3 was reached. At this point, around 10^3^ cells were plated on SD-CAA plates and glycerol stocks containing 20% (w/v) glycerol were prepared and stored at −80 °C. The remaining cells were diluted to an OD_600_ of 0.1–0.2 in 25 mL of SD-CAA and stored at 4 °C until the next experiment was conducted.

### Biosynthesis of the Propargylated Tyrocidine Analogue 1

In vitro biosynthetic reactions were carried out on a 100 μL scale with purified TycA, wt TycB, or M1768A/M1803I TycB and TycC (each at 1 μM) in 100 mM Bis–Tris propane buffer (pH 7.5) containing 100 mM NaCl, 10 mM MgCl_2_, 25 mM ATP, 4 mM TCEP, 1 mM sorbitol, 0.1 units/mL inorganic pyrophosphatase (Sigma I643-100UN), and the nine constituent amino acids of tyrocidine A (1 mM l-Pro, l-Asn, l-Gln, l-Tyr, l-Val, l-Orn, and l-Leu; 2 mM of l-Phe). The standard reaction mixture was supplemented with either one additional equivalent of l-Phe (1 mM) to produce tyrocidine A or four equivalents of pPhe (4 mM) to produce the propargylated analogue **1**. The reaction mixture was incubated at 37 °C in a water bath for 16 h, quenched by addition of 300 μL MeOH, vortexed for 1 min, and centrifuged at full speed for 5 min. The supernatant was transferred into HPLC vials and separated on a Reprosil Gold 120 C18 column (100 × 2 mm, 3 μm) by gradient elution (solvent A: water +0.1% TFA and solvent B: acetonitrile +0.1% TFA; 0–0.5 min = 5% solvent B, 0.5–1.5 min gradient to 55% solvent B, 1.5–5.5 min gradient to 100% B, 5.5–6.6 min hold at 100% solvent B, 6.6–7.0 min equilibrate to 5% solvent B, 7.0–8.0 min hold at 5% solvent B; flow rate 0.75 mL/min, column chamber: 35 °C). The biosynthetic reactions were performed in duplicate with three independent batches of protein.

## Supporting Information Available

Detailed materials and methods, including sequence alignments and cloning strategies; FACS data and sequence alignment of TycB2 variants; protein production, purification, and SDS-PAGE analysis; MS/MS data for the biosynthesized tyrocidine analogue; and DNA and protein sequences of the engineered NRPS modules (PDF)
