# Supplementary material for: High-Throughput Engineering of Nonribosomal Extension Modules
Source: ACS Chem Biol. 2023 Nov 20;18(12):2516–23. doi: 10.1021/acschembio.3c00506 (PMC10728897; doi:10.1021/acschembio.3c00506)
Supplement: Supplementary file 1 — cb3c00506_si_001.pdf [file cb3c00506_si_001.pdf]

Supplementary Information

High-Throughput engineering of nonribosomal extension modules

Anna Camus, Maximilian Gantz and Donald Hilvert*

Laboratory of Organic Chemistry, ETH Zurich, 8093 Zurich, Switzerland

*Correspondence to: hilvert@org.chem.ethz.ch

Table of Contents

Materials and methods 2

Molecular cloning 2

Production of tyrocidine synthetase proteins in *E. coli* 5

Protein purification 5

Supplementary figures 7

Protein sequences 14

DNA sequences 17

References 27

Materials and methods

All chemicals were purchased from Sigma Aldrich and enzymes from NEB unless otherwise stated. Primers (Table S1) were synthesized by Microsynth AG (Switzerland). Propargyl glycine was obtained from Bachem, whereas 4-propargyloxy phenylalanine was synthesized as described by Kries et al.[1] Media and buffer components, kits, and enzymes were used as received from specified commercial suppliers. Expression media and buffers were prepared using purified H_2O (Nanopure system, Barnstead).

Molecular cloning

The plasmids pSU18His-TycA and pTrc99a-TycB1, which respectively encode the C-terminally His_6-tagged TycA protein and the first module of TycB, were obtained from Grünewald et al.[2] Detailed descriptions of the construction of plasmids pSU18His-TycA, pSU18His-$TycA_{pPhe}$, pTrc99a-TycB1$SrfTE_{P26G}$, pTrc99a-TycB, pTrc99a-TycC, and pMG211-Sfp were reported previously.[1,3,4] Plasmid pCT302[5] was used for all yeast experiments but a NheI restriction site was replaced with an NdeI restriction site; the XhoI restriction site was also moved upstream of the c-myc tag, and all other XhoI sites were removed.[6] The cloning protocol for generating pCTRB-TycA A-T and pCTRB- W227S TycA A-T, which contains the W227S mutation, was previously described.[3]

PCRs were conducted using Phusion HF Polymerase (NEB) and GC buffer according to the supplier's protocol (10 µL GC buffer 50-100 ng DNA template, 0.5 µM primer, 0.2 mM dNTPs and 0.5 µL Phusion Polymerase in 50 µL total volume). PCR products were purified by 1% agarose gel electrophoresis and isolated using the Zymoclean™ Gel DNA Recovery Kit (Zymo Research). The resulting PCR products were digested with the appropriate restriction enzymes in CutSmart Buffer at 37 °C for 1 h followed by a purification step using DNA Clean & Concentrator-5 Kit (Zymo Research). The restriction digest of the vector backbone (pSU18-His or pTr99a or pCTRB) vectors was conducted as described for the inserts but the incubation time was increased to 4 h. The vector backbone was also gel-purified and extracted with the Zymoclean™ Gel DNA Recovery Kit (Zymo Research). The ligation of digested PCR products and vector backbone (at a 6:1 ratio, 20-60 ng vector backbone) was performed at room temperature for 15 min using T4 DNA ligase. The ligation product was purified using the DNA Clean & Concentrator-5 Kit, and the plasmid was transformed into electrocompetent *E. coli* HM0079 cells. 0.8 mL SOC medium (0.5% yeast extract, 2% tryptone, 10 mM NaCl, 2.5 mM $MgCl_2$, 10 mM $MgSO_4$, 20 mM glucose) was immediately added to the cells. The transformed cells were grown at 37 °C, 230 rpm for 1 h. 100 µL of the cell solution was plated on LB agar plates supplemented with the appropriate antibiotic (sodium ampicillin for pTrc99a and chloramphenicol for pSU18-His). 5 mL LB pre-cultures containing sodium ampicillin (100 ug/mL) or chloramphenicol (30 µg/mL) were inoculated with a single *E. coli* colony and incubated at 37 °C, 230 rpm overnight. A 1.5 mL culture was centrifuged at 21,000 x *g*. Plasmids were extracted using the ZR Plasmid Miniprep Pure kit (Zymo Research) and sequenced at Microsynth AG (Balgach Switzerland).

Table S1: Primer list

Primer	Primer sequence 5' to 3'	Application
G79	TGCTCCTGTTGCAGGCTTTCC	Cloning TycB variant
G80	AAAGCCTGCAACAGGAGC	Cloning TycB variant
G103	GAGTGCCTGGTGGCCACCTACCTGCGAGATACAC	Cloning TycC variant
G112	GCAAAGATCGCTCGAGATG	Cloning TycB variant
G114	CTCTTTCATTGAGCTCCC	Cloning TycB variant
G150	AACATATCCGACACCGATGCGTC	Cloning TycB variant
G151	ATCGGTGTCGGATATGTTTGGC	Cloning TycB variant
P4n	TGGTGGTGGTTCTCATATGCACTTCAACGACACGGCC	Cloning TycB variant
P6n	ATAAGCTTTTGTTCCTCGAGAATGGCGGCGCCGGCT	Cloning TycB variant
P16n	TGGTGGTGGTTCTCATATGTTCAACGGTGCGCACAAAG	Cloning TycC variant
P18n	ATAAGCTTTTGTTCCTCGAGGATCGGCACATACGTCTC	Cloning TycC variant
P30	CTCGAGGAACAAAAGCTTATT	Cloning TycC variant
P43	CATATGAGAACCACCACCA	General cloning
P44	GCCTTCGATGCCTTTGCC**NNK**ACTTTCTTTACGTTGATTG	Library cloning
P45	GGCAAAGGCATCGAAGGC	Library cloning
P48	GCTCCATCCAGGCAGTC**NNK**CTCGGGGGCGAAAAGC	Library cloning
P49	GACTGCCTGGATGGAGC	Library cloning
P50	CGACGGAGAGCAGCGTC**NNK**GCCACCTACCTGCGAGA	Library cloning
P51	GACGCTGCTCTCCGTCG	Library cloning
P64	TGGTGGTGGTTCTCATATGTTCAACGATACGCACAGAG	Cloning TycB variant
P65	ATA AGC TTT TGT TCC TCG AGG ACA CCC TGC TCG CTC TC	Cloning TycB variant
P87	CCTTTGACGCTTTCGTT**NNK**TCCTTCTTTACGCCTGTGC	Library cloning
P88	AACGAAAGCGTCAAAGG	Library cloning
P89	CGATCTGGTCATCGTCAAC**NNK**TACGGCCCGACAGAAAG	Library cloning
P90	GTTGACGATGACCAGATCG	Library cloning
P91	CGACAGAAAGCAGTGTC**NNK**GCCACCTGGCAGCGC	Library cloning
P92	GACACTGCTTTCTGTCG	Library cloning
P97	TGGTGGTGGTTCTCATATGGAACAGGCAGCCGGCG	Cloning TycB variant
P101	TGGTGGTGGTTCTCATATGGCCAAAGGGAATGTCTTCTCG	Cloning TycC variant
P104	GCTGCTGGTGGCGTTCCTGGATACGCACAGAGAATACC	Cloning TycB variant
P105	GAACGCCACCAGCAGC	Cloning TycB variant
P106	GGTATCCACCCGCCTGCATGCGTCAGGCTGGACG	Cloning TycB variant
P107	CAGGCGGGTGGATACC	Cloning TycB variant

TycB variants

pCTRB-TycB2 A-T: To generate the plasmid for yeast surface display of the A and T domains of the second module of TycB, two PCR fragments were generated using the primer pairs H32/P43, P4n/P6n and the template pTrc99a-TycB, respectively (Table S1).

TycB2 gene library: To create space to accommodate pPhe, residues L1704, M1768, and M1803 in the active site of the TycB2 A domain were randomized using NNK codons. Initially, five fragments were generated using pTRc99a-TycB as a template and the following primer pairs: H32/P43, P4n/P45, P44/P49, P48/P51 and P50/P6n. In a second step, the first three fragments were assembled using primers H32 and P49 and the last two fragments using primers P48 and P6n. In a third step, the two assembled products were connected by overlap PCR using H32 and P6n (Table S1).The PCR product was then transformed into freshly prepared electrocompetent yeast cells together with the pCTRB vector backbone as described below.

pCTRB-M1768A/M1803I-TycB2 A-T: this plasmid was isolated from yeast after sorting the TycB2 library (see Sequencing plasmids from homologous recombination or library clones)

pTrc99a-M1768A/M1803I-TycB: To introduce the mutations that confer pPhe specificity into the full-length TycB gene, two fragments were produced by PCR using the primer pairs G112/G79 and G80/G114 and pCTRB-M1768A/M1803I-TycB2 AT and pTrc99a-TycB as templates, respectively (Table 5). The two fragments were subsequently assembled by PCR. The purified and assembled PCR product was digested with XhoI and SacI and incubated for 1 h at 37 °C. The digested insert was purified using the Clean and Concentrator-5 kit (Zymo Research). The purified insert and the pTrc99a vector backbone were ligated using T4 DNA ligase in a ratio of 1:6 at 16 °C for 16 h. The ligated plasmid was purified using the Clean and Concentrator-5 kit (Zymo Research) and transformed by electroporation into electrocompetent HM0079 cells. The cells were resuspended in 800 µL SOC medium which contains 0.5% yeast extract, 2% tryptone, 10 mM NaCl, 2.5 mM $MgCl_2$, 10 mM $MgSO_4$, 20 mM glucose. The suspension was grown for 1 h at 37 °C and plated onto LB-agar plates containing 100 µg/mL ampicillin. The plates were incubated at 37 °C for 16 h. 3 mL LB Miller medium were inoculated with a single colony and incubated at 37 °C, 230 rpm for 16 h. The plasmid was isolated using the ZymoPURE^{TM} Plasmid Miniprep Kit and sequenced by Microsynth AG (Switzerland). A 20% (w/v) glycerol stock of the culture containing the plasmid was prepared and stored at -80 °C.

pCTRB-W2742S-TycB3 A-T: To generate the plasmid for yeast surface display of the A and T domains of the third module of TycB containing the W2742S mutation, three PCR fragments were generated using the primer pairs H32/P43 (template: pCTRB-TycA A-T), P64/G150 (template: pTrc99a-TycB), and G151/P65 (template: pTrc99a-TycB) (Table S1).

pCTRB-TycB3 C-A-T: To generate the plasmid for yeast surface display of the C, A and T domains of the third module of TycB, three PCR fragments were generated using the primer pairs H32/P43 (template: pCTRB-TycA A-T), P97/P65 (template: pTrc99a-TycB), and P30/H33 (template: pCTRB-TycA A-T) (Table S1).

pCTRB-W2742S-TycB3 C-A-T: To generate the plasmid for yeast surface display of the C, A, and T domains of the third module of TycB containing the W2742S mutation, three PCR fragments were generated using the primer pairs H32/P105 (template: pCTRB-TycB3 C-A-T), P104/P107 (template: pTrc99a-W2742S-TycB), and P106/H33 (template: pCTRB-TycB3 C-A-T) (Table S1).

TycC variants

pCTRB-TycC3 A-T: To generate the plasmid for yeast surface display of the A and T domains of the third module of TycC, two PCR fragments were generated using the primer pairs H32/P43 and P16n/P18n and the templates pCTRB-TycA A-T and pTrc99a-TycC, respectively (Table S1).

pCTRB-TycC6 C-A-T: To generate the plasmid for yeast surface display of the C, A and T domains of the last module of TycC, three PCR fragments were generated using the primer pairs H32/P43 (template: pCTRB-TycA AT), P101/G103 (template: pTrc99a-TycC), and P30/H33 (template: pCTRB-TycA AT) (Table S1).

Production of tyrocidine synthetase proteins in *E. coli*

To produce tyrocidine synthetase, the procedure reported by Niquille and Folger et al.[4] was adopted with minor adaptations. The constituent proteins of the synthetase were produced with an N-terminal (TycA) or C-terminal (TycB variants and TycC) His_6-tag in *E. coli* strain HM0079.[2] The cell cultures were supplemented with chloramphenicol (30 µg/mL, PanReac AppliChem) for TycA production or with ampicillin (100 µg/mL, PanReac AppliChem) for TycB and TycC production. Briefly, 5 mL LB Miller broth containing the appropriate antibiotic were inoculated with a single HM0079 colony transformed with the desired plasmid (pSU18His-TycA, pTrc99a-TycB, pTrc99-M1786A/M1803I-TycB or pTrc99a-TycC) and incubated overnight at 37 °C, 230 rpm. The culture was prepared in a 2 L baffled flask, containing 800 mL modified Studier medium.[10] The modified Studier medium is based on LB Miller broth to which $MgSO_4$ (2 mM), glycerol (Acros, 1% (m/v)), Na_2HPO_4 (25 mM), KH_2PO_4 (FisherBio, 25 mM), NH_4Cl (50 mM), and Na_2SO_4 (5 mM) were added. The medium was additionally supplemented with the appropriate antibiotic, inoculated at a 1:500 ratio with the pre-culture and incubated at 37 °C, 190 rpm for 4-6 h until an OD_{600} of around 2 was reached. At this point, protein expression was induced by adding isopropyl β-d-1-thiogalactopyranoside (PanReac AppliChem, 250 µM), and the culture was incubated at 20 °C, 190 rpm for 20 h. The cells were centrifuged at 5,000 x g, 4 h for 20 min and the pellet was stored at -20 °C.

Protein purification

The heterologously expressed tyrocidine synthetase proteins were purified as reported by Niquille and Folger et al.[4] Briefly, to lyse the HM0079 cells containing the protein of interest,

the thawed pellet was resuspended in 30 mL Tris-HCl (50 mM) buffer pH 8.0, supplemented with NaCl (500 mM), and glycerol (10% (w/v). Polymyxin (Apollo, 1 mg/mL), RNase A (AppliChem, 10 µg/mL), DNase (PanReac AppliChem10 µg/mL), and lysozyme (PanReac, 1 mg/ml) were added to increase lysis efficiency. The solution was incubated at 4 °C for 20 min before sonication at 60% amplitude in a Q700 sonicator (Qsonica) equipped with a 1/4 microtip (sonication time: 4 cycles of 30 s each with a 30 s break in between). The solution was centrifuged at 15,000 x g for 20 min at 4 °C and the supernatant containing the soluble protein was applied to pre-equilibrated Ni-NTA columns containing 5 mL Ni-beads (Quiagen). The collected flow-through was added to the Ni-NTA column again. The proteins bound to the Ni-NTA were washed with 40 mL Tris-HCl (50 mM) buffer pH 8.0, containing NaCl (500 mM), imidazole (Apollo Scientific, 20 mM), and glycerol (10% (w/v)). The bound protein was eluted from the column by addition of 10 mL Tris-HCl (50 mM) buffer pH 8.0, supplemented with NaCl (500 mM), imidazole (300 mM), and glycerol (10% (w/v)). The buffer was exchanged to Bis-Tris propane (Apollo, 50 mM) pH 7.25, containing NaCl (100 mM), $MgCl_2$ (10 mM), glycerol (10% (w/v)) using Amicon-Ultra-15 centrifugal filters (Merck, MWCO=10,000). The proteins were aliquoted, flash frozen in liquid nitrogen and stored at -80 °C until further use. Protein concentration was determined from the absorption at 280 nm measured on a Nanodrop 2000 spectrophotometer (Thermo Fisher). Extinction coefficients were calculated by the https://web.expasy.org/protparam/ webtool. Proteins were analyzed on 7.5% SDS-PAGE gels using a Phast system to assess purity.

MS/MS analysis of the propargylated tyrocidine analogue

Aliquots (10 µL) from the quenched biosynthetic reaction mixtures were analyzed on an Agilent 1290 Infinity II liquid chromatography system coupled to a Bruker maXisII-ESI-Q-TOF MS system (Bruker Daltonics) and equipped with an Agilent ZorbaxEclipse Plus C18 column (3.5 µm 3.0 x 50 mm). The mobile phase was a mixture of 0.1% (v/v) formic acid in water and 0.1% (v/v) formic acid in acetonitrile. A linear gradient from 2% to 98% acetonitrile over 10 min was used at a flow rate of 0.6 mL/min. The ESI-TOF mass spectrometer was calibrated routinely for LC-MS in the positive electrospray ionization mode using the Agilent-ESI-TOF tuning mix in the enhanced quadratic algorithmic mode. The offset of each spectrum in the chromatogram was corrected with methylstearate and an Agilent Tunemix-mass 622 (m/z 299.2945 and 622.0290 respectively). The optimized source conditions were 8.0 l/h drying gas (nitrogen 99,99% purity) at a temperature of 180 °C, 1.6 bar nebulizer pressure, capillary and endplate voltages of 4,500 and 500 V, respectively, 12,000 V TOF flight tube voltage, 3,183 V reflection voltage, 1,700 V pusher voltage, and 2,268 V MCP detector voltage. The resolving power of the instrument was around 100.000 at m/z 1375 with 4 Hz spectra rate, depending on the sample concentration and peak width. For LC-MS/MS experiments (collision-induced dissociation; CID), the "Auto-MSMS-Mode" with an automatic precursor selection (5 Highest MS Signals) was used; isolation width m/z 3 at m/z 1375) and variable collision energy (20-90 eV; ISCID 0.0 eV) with nitrogen (99.99%) as collision gas (flow rate 28%). Data Analysis 5.1 (Built 201.2.4019) (64bit) software (Bruker Daltonics, Germany) was used for data processing.

Supplementary figures

a

A domain	"223"	"224"	"227"	"266"	"287"	"289"	"310"	"318"	"319"	"506"
TycA	D	A	W	T	I	A	A	I	C	K
TycB1	D	V	Q	S	I	A	H	H	V	K
TycB2	D	A	L	V	M	G	A	V	M	K
TycB3	D	A	W	T	I	A	G	V	C	K
TycC1	D	L	T	K	I	G	E	V	G	K
TycC2	D	A	W	Q	F	G	L	I	D	K
TycC3	D	A	L	T	T	G	E	V	V	K
TycC4	D	A	F	W	I	G	G	T	F	K
TycC5	D	V	G	E	I	G	S	I	D	K
TycC6	D	G	A	Y	T	G	E	V	V	K

b

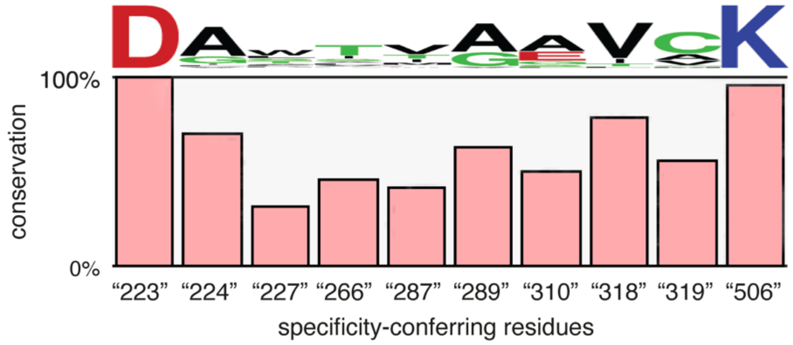

Figure S1: (a) Sequence alignment of the specificity determining residues of all ten A domains of tyrocidine synthetase. Dark red indicates high conservation and dark blue high variability. (b) Conservation of the specificity determining residues of 27 A domains, including TycA, TycB2, TycB3 and TycC3 from panel (a), that activate aromatic amino acids (twelve specific for Tyr, eleven for Phe, three for Trp, and one for phenyl glycine).[13,14] TycA numbering is used. The sequence logo above the bar graph indicates the preferred residue(s).

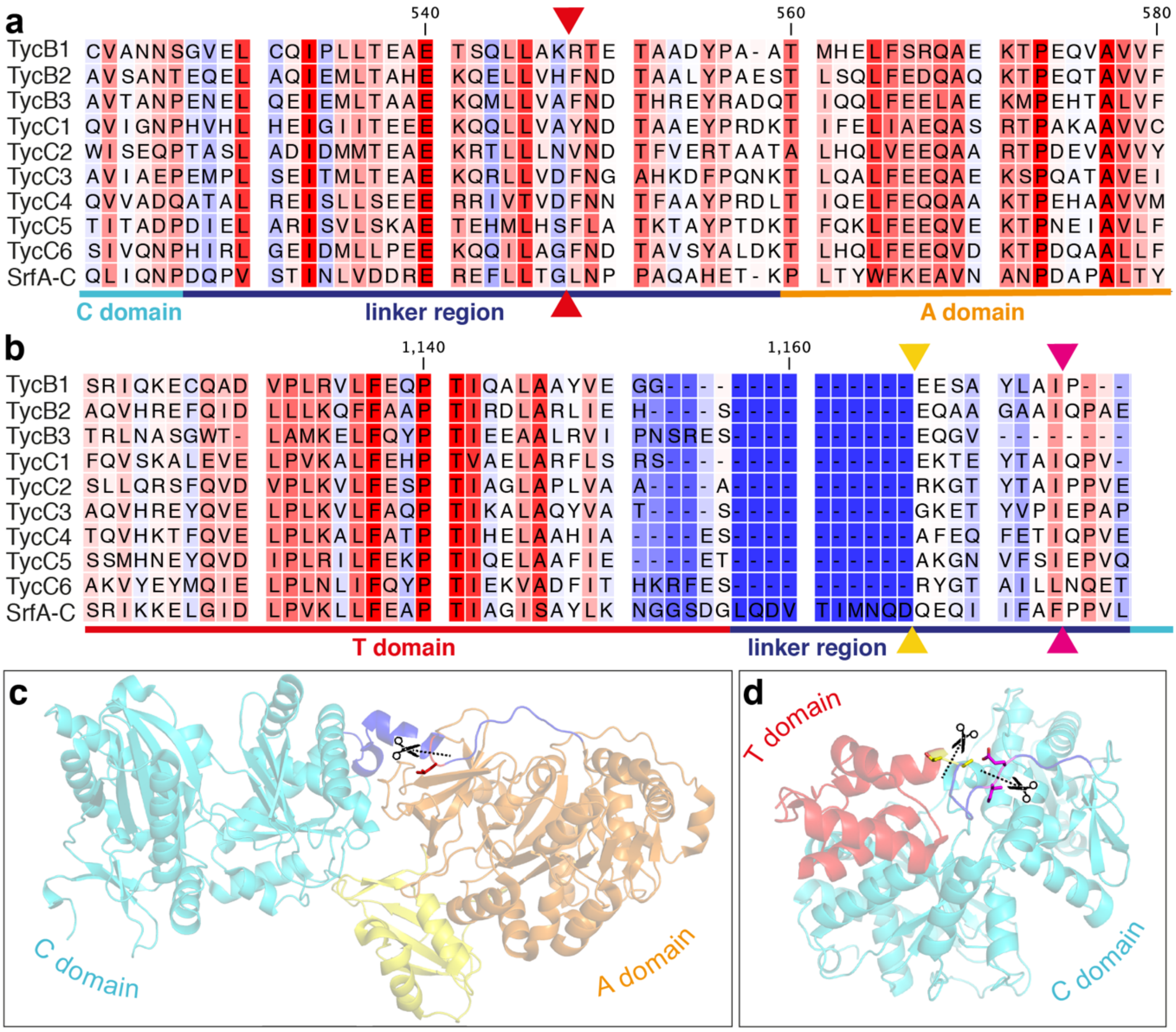

Figure S2. Cleavage sites for excising NRPS elongation modules for functional display on yeast. (a) Sequence alignment of the linker regions connecting the C and A domains of all TycB and TycC modules with structurally characterized SrfA-C, which served as a reference. The N-terminal excision site selected for the A-T constructs is indicated by red triangles. Dark red indicates high conservation, whereas dark blue indicates high variability. (b) The C-terminal excision site for the A-T and C-A-T constructs (magenta triangles) and the N-terminal excision site for C-A-T constructs (yellow triangles). (c) Crystal structure of SrfA-C (PDB: 2VSQ, residues 398-510),[11] showing the N-terminal excision site for the A-T constructs (red). The C domain is colored cyan, the A domain orange/yellow, and linker regions dark blue. (d) Crystal structure of the TycC5-TycC6 T-C bidomain (PDB: 2JGP)[12] showing the N-terminal excision site for C-A-T constructs (yellow) and C-terminal (magenta) excision site for A-T and C-A-T constructs. The T and C domains are colored red and cyan, respectively.

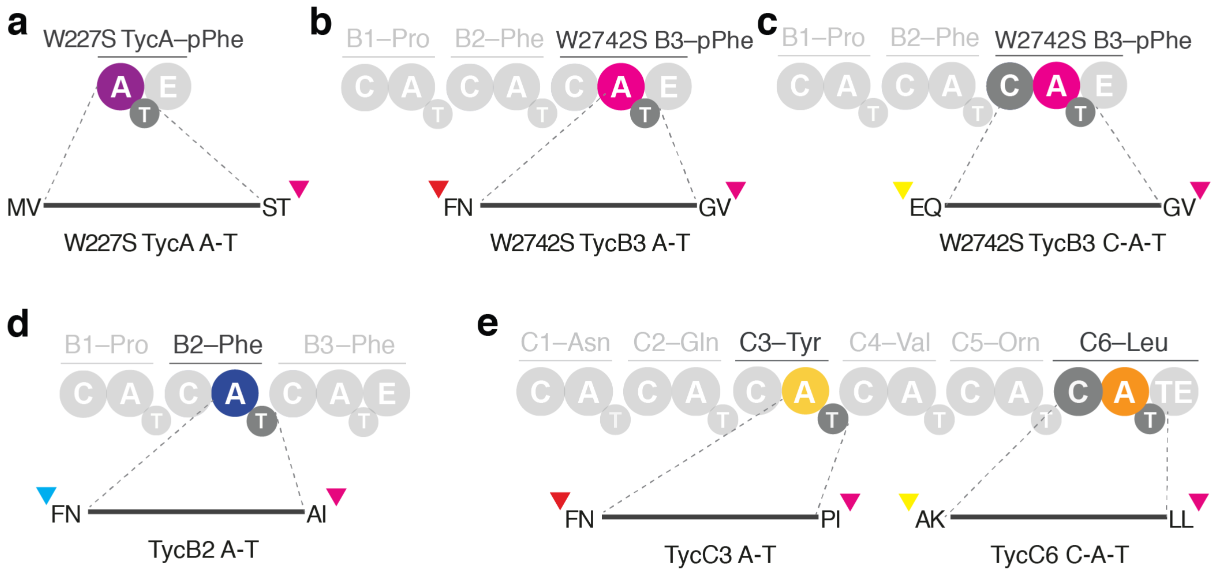

Figure S3. Tyrocidine synthetase domains/modules chosen for yeast display, showing their native context within the megaenzyme. The segment below the synthetase indicates the N- and C-terminal amino acids of the display constructs with triangles, colored as in Figure S2, indicating the cleavage sites chosen. (a) The previously described TycA A-T bidomain.[3] Its A domain (purple) contained the W227S mutation that confers pPhe specificity. The corresponding (b) A-T and (c) C-A-T constructs from TycB3 are shown. The A domain of TycB3 (magenta) contained the W2742S mutation to accommodate pPhe. (d) The analogous A-T construct from TycB2 was engineered in high-throughput to switch its substrate specificity from L-Phe to pPhe. (e) The A-T construct from TycC3 (yellow) and the C-A-T construct from TycC6 (orange) are illustrated.

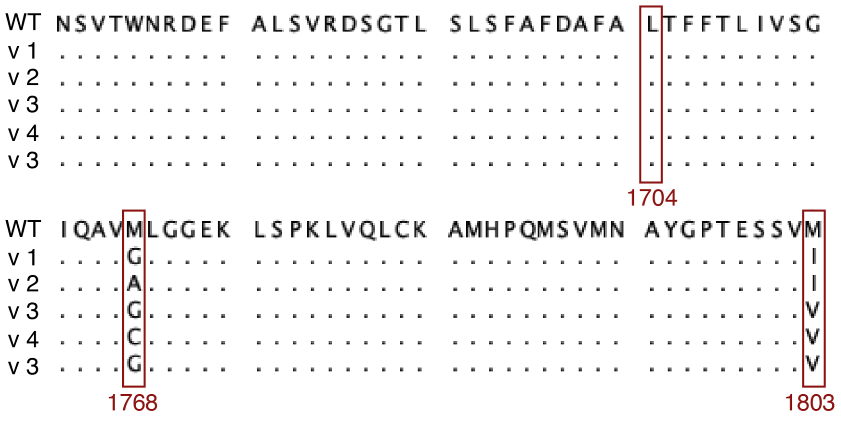

Figure S4: TycB2 mutations that confer activity with pPhe.

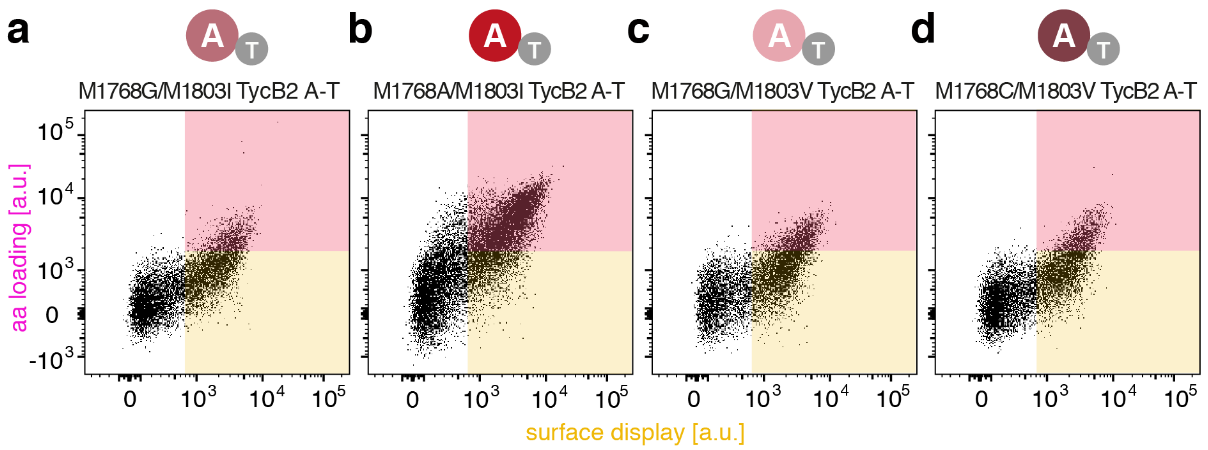

Figure S5: The activity of TycB2 variants 1 (a), 2 (b), 3 (c) and 4 (d) displayed on the surface of yeast. Assays were performed with 0.1 mM ATP and 0.5 mM pPhe in the presence of 0.5 mM Phe as a competitive inhibitor. The fraction of cells in the pink quadrant correlates with the ability of the displayed A domain to activate pPhe and transfer it to the ppan cofactor of the T domain tethered to the cell surface.

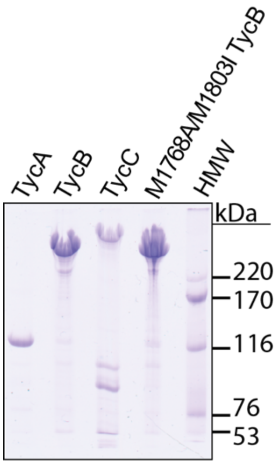

Figure S6: 7.5% SDS-PAGE gel showing purified His_6-tagged TycA, TycB, TycC and the variant M1768A/M1803I TycB produced in *E. coli* HM007. HMW= High molecular weight ladder (GE).

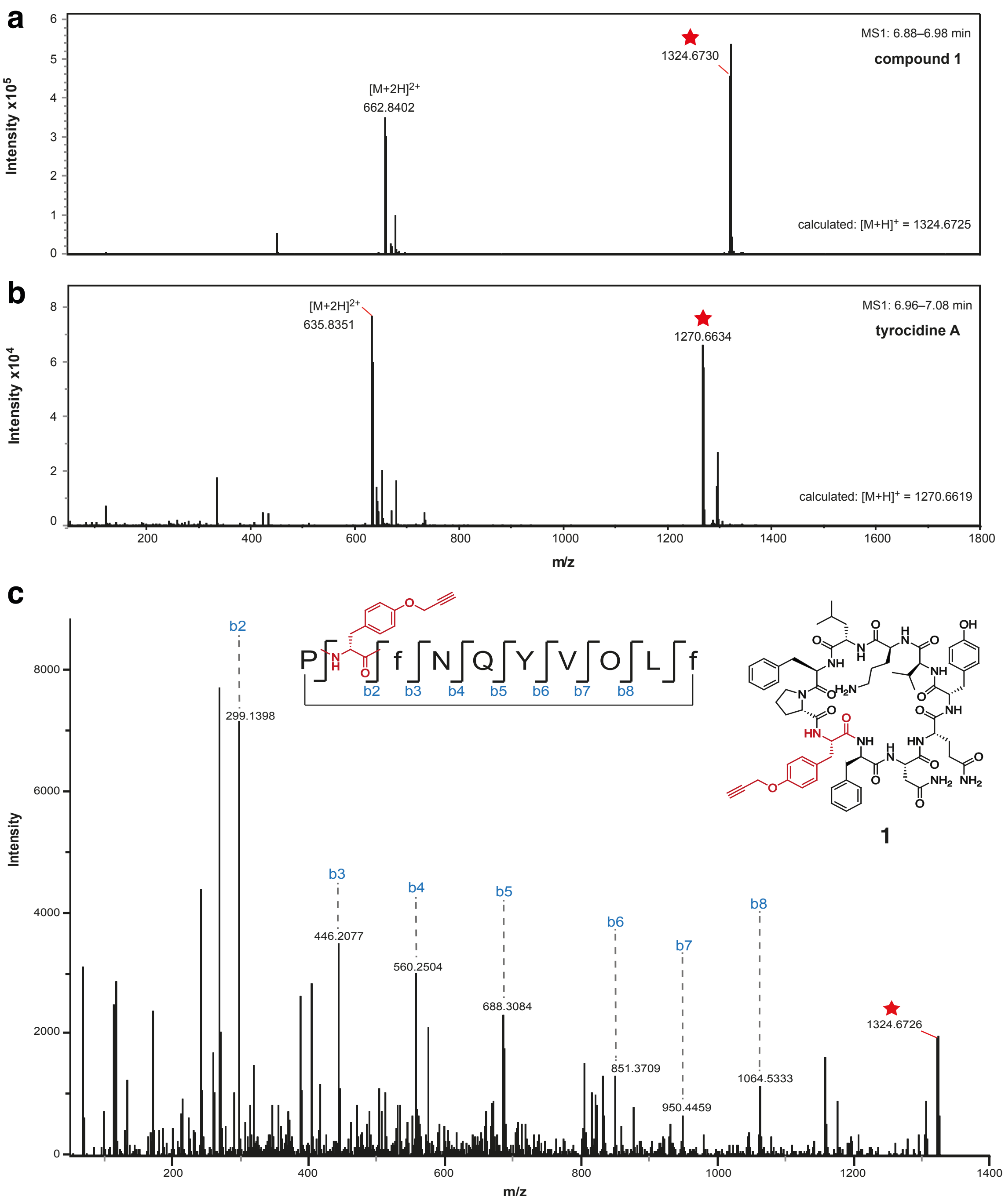

Figure S7: MS/MS characterization of the products of the in vitro biosynthetic reaction catalyzed by TycA, M1768A/M1803I TycB and TycC in the presence of pPhe. (a) The selected mass of the major product that elutes at a retention time of 6.88–6.98 (see Fig. 5 in the main text) is consistent with the propargyloxy-tyrocidine derivative 1 (calculated $[M+H]^+$ 1324.6725 Da; found 1324.6730 Da). (b) The side product that elutes at a retention time of 6.96–7.08 min is wildtype tyrocidine A (calculated $[M+H]^+$ 1270.6619; found 1270.6634 Da). (c) MS2 spectrum of the major product confirms its assignment to structure 1, with the noncanonical pPhe incorporated site-specifically as the third residue in the natural product analogue. The observed fragmentation masses (b-ions) are shown in blue below the single letter code of the sequence, the sites of fragmentation are marked with dashed lines, and the parent ion is highlighted with a red star. O stands for Orn and f for D-Phe; all other capital single letters refer to L-amino acids.

Protein sequences

Aga-HA-W2742S-TycB3-A-T-c-myc (protein sequence displayed on yeast, bold amino acid marks start of NRPS protein and highlighted in blue is W2742S mutation)

MQLLRCFSIFSVIASVLAQELTTICEQIPSPTLESTPYSLSTTTILANGKAMQGVFEYYKSVTFVSNCGSHPSTTSKGSP
INTQYVFKDNSSTIEGRYPYDVPDYALQASGGGGSGGGGSGGGGSHM**F**NDTHREYRADQTIQQLFEELAEKMPEH
TALVFEEKRMSFRELNERANQLAAVLREKGVGPAQIVALLVERSAEMVIATLATLKAGGAFLPVDPDYPEERIRYM
LEDSQAKLVVTHAHLLHKVSSQSEVVDVDDPGSYATQTDNLPCANTPSDLAYIIYTSGTTGKPKGVMLEHKGVAN
LQAVFAHHLGVTPQDRAGHFASISFDASV**S**DMFGPLLSGATLYVLSRDVINDFQRFAEYVRDNAITFLTLPPTYAIY
LEPEQVPSLRTLITAGSASSVALVDKWKEKVTYVNGYGPTESTVCATLWKAKPDEPVETITIGKPIQNTKLYIVDDQ
LQLKAPGQMGELCISGLSLARGYWNRPELTAEKFVDNPFVPGTKMYRTGDLARWLPDGTIEYLGRIDHQVKIRGH
RVELGEVESVLLRYDTVKEAAAITHEDDRGQAYLCAYYVAEGEATPAQLRAYMENELPNYMVPAFFIQLEKMPLT
PNDKIDRKALPKPNQEENRTEQYAAPQTELEQLLAGIWADVLGIKQVGTQDNFFELGGDSIKAIQVSTRLNASGWT
LAMKELFQYPTIEEAALRVIPNSRESEQGVLEEQKLISEEDL

Aga-HA-W2742S-TycB3-C-A-T-c-myc (protein sequence displayed on yeast, bold amino acid marks start of NRPS protein and highlighted in blue is W2742S mutation)

MQLLRCFSIFSVIASVLAQELTTICEQIPSPTLESTPYSLSTTTILANGKAMQGVFEYYKSVTFVSNCGSHPSTTSKGSP
INTQYVFKDNSSTIEGRYPYDVPDYALQASGGGGSGGGGSGGGGSHM**E**QAAGAAIQPAEPQAYYPVSSAQQRMY
LLHQLEGAGISYNTPGIIMLEGKLDREQLANALQALVDRHDILRTSFEMVGDELVQKIHDRVAVNMEYVTAEEQQI
DDLFHAFVRPFDLSVPPLLRMSLVKLADERHLLLYDMHHIAADAASITILFDELAELYQGRELPEMRIQYKDFAVW
QKALHESDAFKQQEAYWLSTFAGNITAVDVPTDFPRPAVKSFAGGQVTLSMDQELLSALHELAAHTNTTLFMVLL
AAYNVLLAKYAGQDDIIVGTPISGRSRAELAPVVGMFVHTLAIRNKPTAEKTFKQFLQEVKQNALDAFDHQDYPFE
SLVEKLGIPRDPGRNPLFDTMFILQNDELHAKTLDQLVYRPYESDSALDVAKFDLSFHLTERETDLFLRLEYCTKLF
KQQTVERMAHHFLQILRAVTANPENELQEIEMLTAAEKQMLLVAFNDTHREYRADQTIQQLFEELAEKMPEHTAL
VFEEKRMSFRELNERANQLAAVLREKGVGPAQIVALLVERSAEMVIATLATLKAGGAFLPVDPDYPEERIRYMLED
SQAKLVVTHAHLLHKVSSQSEVVDVDDPGSYATQTDNLPCANTPSDLAYIIYTSGTTGKPKGVMLEHKGVANLQA
VFAHHLGVTPQDRAGHFASISFDASV**S**DMFGPLLSGATLYVLSRDVINDFQRFAEYVRDNAITFLTLPPTYAIYLEPE
QVPSLRTLITAGSASSVALVDKWKEKVTYVNGYGPTESTVCATLWKAKPDEPVETITIGKPIQNTKLYIVDDQLQLK
APGQMGELCISGLSLARGYWNRPELTAEKFVDNPFVPGTKMYRTGDLARWLPDGTIEYLGRIDHQVKIRGHRVEL
GEVESVLLRYDTVKEAAAITHEDDRGQAYLCAYYVAEGEATPAQLRAYMENELPNYMVPAFFIQLEKMPLTPNDK
IDRKALPKPNQEENRTEQYAAPQTELEQLLAGIWADVLGIKQVGTQDNFFELGGDSIKAIQVSTRLNASGWTLAMK
ELFQYPTIEEAALRVIPNSRESEQGVLEEQKLISEEDL

Aga-HA-TycC3-A-T-c-myc (protein sequence displayed on yeast, bold amino acid marks start of NRPS protein)

MQLLRCFSIFSVIASVLAQELTTICEQIPSPTLESTPYSLSTTTILANGKAMQGVFEYYKSVTFVSNCGSHPSTTSKGSP
INTQYVFKDNSSTIEGRYPYDVPDYALQASGGGGSGGGGSGGGGSHM**F**NGAHKDFPQNKTLQALFEEQAEKSPQA
TAVEISGQPLSYQELNERANQLAATLRERGVQPDQPVGIMANRSVEMVVGILAILKAGGAYVPIDPEYPEERVAYM
LTDCQARLVLTQKHLGAKLGSSVTAECLYLDDESNYGVHRSNLQPINTASDLAYIIYTSGTTGKPKGVMVEHRGIV
NNVLWKKAEYQMKVGDRSLLSLSFAFDAFVLSFFTPVLSGATVVLAEDEEAKDPVSLKKLIAASRCTLMTGVPSLF
QAILECSTPADIRPLQTVTLGGEKITAQLVEKCKQLNPDLVIVNEYGPTESSVVATWQRLAGPDAAITIGRPIANTSL
YIVNQYHQLQPIGVVGEICIGGRGLARGYWNKPALTEEKFVSHPFAAGERMYKTGDLGKWLPDGTIEYIGRIDEQV
KVRGYRIEIGEIESALLAAEKLTAAVVVVYEDQLGQSALAAYFTADEQLDVTKLWSHLSKRLPSYMIPAHFVQLDQ
LPLTPNGKVDKKALPKPEGKPVTEAQYVAPTNAVESKLAEIWERVLGVSGIGILDNFFQIGGHSLKAMAVAAQVHR
EYQVELPLKVLFAQPTIKALAQYVATSGKETYVPILEEQKLISEEDL

Aga-HA-TycC6-C-A-T-c-myc (protein sequence displayed on yeast, bold amino acid marks start of NRPS protein)

MQLLRCFSIFSVIASVLAQELTTICEQIPSPTLESTPYSLSTTTILANGKAMQGVFEYYKSVTFVSNCGSHPSTTSKGSP
INTQYVFKDNSSTIEGRYPYDVPDYALQASGGGGSGGGGSGGGGSHM**A**KGNVFSIEPVQKQAYYPVSSAQKRMYI
LDQFEGVGISYNMPSTMLIEGKLERTRVEAAFQRLIARHESLRTSFAVVNGEPVQNIHEDVPFALAYSEVTEQEARE
LVSSLVQPFDLEVAPLIRVSLLKIGEDRYVLFTDMHHSISDGVSSGILLAEWVQLYQGDVLPELRIQYKDFAVWQQE
FSQSAAFHKQEAYWLQTFADDIPVLNLPTDFTRPSTQSFAGDQCTIGAGKALTEGLHQLAQATGTTLYMVLLAAY
NVLLAKYAGQEDIIVGTPITGRSHADLEPIVGMFVNTLAMRNKPQREKTFSEFLQEVKQNALDAYGHQDYPFEELV
EKLAIARDLSRNPLFDTVFTFQNSTEEVMTLPECTLAPFMTDETGQHAKFDLTFSATEEREEMTIGVEYSTSLFTRET
MERFSRHFLTIAASIVQNPHIRLGEIDMLLPEEKQQILAGFNDTAVSYALDKTLHQLFEEQVDKTPDQAALLFSEQSL
TYSELNERANRLARVLRAKGVGPDRLVAIMAERSPEMVIGILGILKAGGAYVPVDPGYPQERIQYLLEDSNAALLLS
QAHLLPLLAQVSSELPECLDLNAELDAGLSGSNLPAVNQPTDLAYVIYTSGTTGKPKGVMIPHQGIVNCLQWRRDE
YGFGPSDKALQVFSFAFDGFVASLFAPLLGGATCVLPQEAAAKDPVALKKLMAATEVTHYYGVPSLFQAILDCSTT
TDFNQLRCVTLGGEKLPVQLVQKTKEKHPAIEINNEYGPTENSVVTTISRSIEAGQAITIGRPLANVQVYIVDEQHHL
QPIGVVGELCIGGAGLARGYLNKPELTAEKFVANPFRPGERMYKTGDLVKWRTDGTIEYIGRADEQVKVRGYRIEI
GEIESAVLAYQGIDQAVVVARDDDATAGSYLCAYFVAATAVSVSGLRSHLAKELPAYMIPSYFVELDQLPLSANG
KVDRKALPKPQQSDATTREYVAPRNATEQQLAAIWQEVLGVEPIGITDQFFELGGHSLKATLLIAKVYEYMQIELPL
NLIFQYPTIEKVADFITHKRFESRYGTAILLLEEQKLISEEDL

Aga-HA-TycB2-A-T-c-myc (protein sequence displayed on yeast, bold amino acid marks start of NRPS protein and highlighted in red are target sites for mutagenesis)

MQLLRCFSIFSVIASVLAQELTTICEQIPSPTLESTPYSLSTTTILANGKAMQGVFEYYKSVTFVSNCGSHPSTTSKGSP
INTQYVFKDNSSTIEGRYPYDVPDYALQASGGGGSGGGGSGGGGSHM**F**NDTAALYPAESTLSQLFEDQAQKTPEQ
TAVVFGDKRLTYRELNERANQLAHTLRAKGVQAEQSVGIMAQRSLEMAIGIIAILKAGGAYVPIDPDYPNERIAYM
LEDCEARLVLTQQQLAEKMTANVECLYLDEEGSYSPQTENIEPIHTAADLAYIIYTSGTTGRPKGVMVEHRGIVNSV
TWNRDEFALSVRDSGTLSLSFAFDAFA**L**TFFTLIVSGSTVVLMPDHEAKDPIALRNLIAAWECSYVVFVPSMFQAIL
ECSTPADIRSIQAV**M**LGGEKLSPKLVQLCKAMHPQMSVMNAYGPTESSV**M**ATYLRDTQPDQPITIGRPIANTAIYIV
DQHHQLLPVGVVGEICIGGHGLARGYWKKPELTAEKFVANPAVPGERMYKTGDLGRWLHDGTIDFIGRVDDQIKV
RGYRIEVGEIEAVLLAYDQTNEAIVVAYQDDRGDSYLAAYVTGKTAIEESELRAHLLRELPAYMVPTYLIQLDAFPL
TPNGKVDRKALPKPEGKPATGAAYVAPATEVEAKLVAIWENALGISGVGVLDHFFELGGHSLKAMTVVAQVHRE
FQIDLLLKQFFAAPTIRDLARLIEHSEQAAGAAILEEQKLISEEDL

M1768A/M1803I-TycB-His (best protein variant isolated from the library for activation of *4-propargyloxy phenylalanine*. The mutated residues are highlighted in red)

MSVFSKEQVQDMYALTPMQEGMLFHALLDQEHNSHLVQMSISLQGDLDVGLFTDSLHVLVERYDVFRTLFLYEK
LKQPLQVVLKQRPIPIEFYDLSACDESEKQLRYTQYKRADQERTFHLAKDPLMRVALFQMSQHDYQVIWSFHHILM
DGWCFSIIFDDLLAIYLSLQNKTALSLEPVQPYSRFINWLEKQNKQAALNYWSDYLEAYEQKTTLPKKEAAFAKAF
QPTQYRFSLNRTLTKQLGTIASQNQVTLSTVIQTIWGVLLQKYNAAHDVLFGSVVSGRPTDIVGIDKMVGLFINTIPF
RVQAKAGQTFSELLQAVHKRTLQSQPYEHVPLYDIQTQSVLKQELIDHLLVIENYPLVEALQKKALNQQIGFTITAV
EMFEPTNYDLTVMVMPKEELAFRFDYNAALFDEQVVQKLAGHLQQIADCVANNSGVELCQIPLLTEAETSQLLAK
RTETAADYPAATMHELFSRQAEKTPEQVAVVFADQHLTYRELDEKSNQLARFLRKKGIGTGSLVGTLLDRSLDMI
VGILGVLKAGGAFVPIDPELPAERIAYMLTHSRVPLVVTQNHLRAKVTTPTETIDINTAVIGEESRAPIESLNQPHDLF
YIIYTSGTTGQPKGVMLEHRNMANLMHFTFDQTNIAFHEKVLQYTTCSFDVCYQEIFSTLLSGGQLYLITNELRRHV
EKLFAFIQEKQISILSLPVSFLKFIFNEQDYAQSFPRCVKHIITAGEQLVVTHELQKYLRQHRVFLHNHYGPSETHVVT
TCTMDPGQAIPELPPIGKPISNTGIYILDEGLQLKPEGIVGELYISGANVGRGYLHQPELTAEKFLDNPYQPGERMYR
TGDLARWLPDGQLEFLGRIDHQVKIRGHRIELGEIESRLLNHPAIKEAVVIDRADETGGKFLCAYVVLQKALSDEEM
RAYLAQALPEYMIPSFFVTLERIPVTPNGKTDRRALPKPEGSAKTKADYVAPTTELEQKLVAIWEQILGVSPIGIQDH
FFTLGGHSLKAIQLISRIQKECQADVPLRVLFEQPTIQALAAYVEGGEESAYLAIPQAEPQAYYPVSSAQKRMLILNQ
LDPHSTVYNLPVAMILEGTLDKARLEHAISNLVARHESLRTSFHTINGEPVSRIHEQGHLPIVYLETAEEQVNEVILG
FMQPFDLVTAPLCRVGLVKLAENRHVLIIDMHHIISDGVSSQLILNDFSRLYQNKALPEQRIHYKDFAVWEKAWTQ
TTDYQKQEKYWLDRFAGEIPVLNLPMDYPRPAVQSFEGERYLFRTEKQLLESLQDVAQKTGTTLYMVLLAAYHVL
LSKYSGQDDVMIGTVTAGRVHPDTESMTGMFVNTLAMRNQSAPTKTFRQFLLEVKDNTLAAFEHGQYPFEELVEK
LAIQRNRSRNPLFDTLFILQNMDADLIELDGLTVTPYVPEGEVAKFDLSLEASENQAGLSFCFEFCTKLFARETIERM

SLHYLQILQAVSANTEQELAQIEMLTAHEKQELLVHFNDTAALYPAESTLSQLFEDQAQKTPEQTAVVFGDKRLTY
RELNERANQLAHTLRAKGVQAEQSVGIMAQRSLEMAIGIIAILKAGGAYVPIDPDYPNERIAYMLEDCEARLVLTQ
QQLAEKMTANVECLYLDEEGSYSPQTENIEPIHTAADLAYIIYTSGTTGRPKGVMVEHRGIVNSVTWNRDEFALSV
RDSGTLSLSFAFDAFALTFFTLIVSGSTVVLMPDHEAKDPIALRNLIAAWECSYVVFVPSMFQAILECSTPADIRSIQA
V**A**LGGEKLSPKLVQLCKAMHPQMSVMNAYGPTESSV**I**ATYLRDTQPDQPITIGRPIANTAIYIVDQHHQLLPVGVV
GEICIGGHGLARGYWKKPELTAEKFVANPAVPGERMYKTGDLGRWLHDGTIDFIGRVDDQIKVRGYRIEVGEIEAV
LLAYDQTNEAIVVAYQDDRGDSYLAAYVTGKTAIEESELRAHLLRELPAYMVPTYLIQLDAFPLTPNGKVDRKALP
KPEGKPATGAAYVAPATEVEAKLVAIWENALGISGVGVLDHFFELGGHSLKAMTVVAQVHREFQIDLLLKQFFAA
PTIRDLARLIEHSEQAAGAAIQPAEPQAYYPVSSAQQRMYLLHQLEGAGISYNTPGIIMLEGKLDREQLANALQALV
DRHDILRTSFEMVGDELVQKIHDRVAVNMEYVTAEEQQIDDLFHAFVRPFDLSVPPLLRMSLVKLADERHLLLYD
MHHIAADAASITILFDELAELYQGRELPEMRIQYKDFAVWQKALHESDAFKQQEAYWLSTFAGNITAVDVPTDFPR
PAVKSFAGGQVTLSMDQELLSALHELAAHTNTTLFMVLLAAYNVLLAKYAGQDDIIVGTPISGRSRAELAPVVGM
FVHTLAIRNKPTAEKTFKQFLQEVKQNALDAFDHQDYPFESLVEKLGIPRDPGRNPLFDTMFILQNDELHAKTLDQL
VYRPYESDSALDVAKFDLSFHLTERETDLFLRLEYCTKLFKQQTVERMAHHFLQILRAVTANPENELQEIEMLTAA
EKQMLLVAFNDTHREYRADQTIQQLFEELAEKMPEHTALVFEEKRMSFRELNERANQLAAVLREKGVGPAQIVAL
LVERSAEMVIATLATLKAGGAFLPVDPDYPEERIRYMLEDSQAKLVVTHAHLLHKVSSQSEVVDVDDPGSYATQT
DNLPCANTPSDLAYIIYTSGTTGKPKGVMLEHKGVANLQAVFAHHLGVTPQDRAGHFASISFDASVWDMFGPLLS
GATLYVLSRDVINDFQRFAEYVRDNAITFLTLPPTYAIYLEPEQVPSLRTLITAGSASSVALVDKWKEKVTYVNGYG
PTESTVCATLWKAKPDEPVETITIGKPIQNTKLYIVDDQLQLKAPGQMGELCISGLSLARGYWNRPELTAEKFVDNP
FVPGTKMYRTGDLARWLPDGTIEYLGRIDHQVKIRGHRVELGEVESVLLRYDTVKEAAAITHEDDRGQAYLCAYY
VAEGEATPAQLRAYMENELPNYMVPAFFIQLEKMPLTPNDKIDRKALPKPNQEENRTEQYAAPQTELEQLLAGIW
ADVLGIKQVGTQDNFFELGGDSIKAIQVSTRLNASGWTLAMKELFQYPTIEEAALRVIPNSRESEQGVVEGEIALTPI
QKWFFANNFTDRHHWNQAVMLFREDGFDEGLVRQAFQQIVEHHDALRMVYKQEDGAIKQINRGLTDERFRFYSY
DLKNHANSEARILELSDQIQSSIDLEHGPLVHVALFATKDGDHLLVAIHHLVVDGVSWRILFEDFSSAYSQALHQQE
IVLPKKTDSFKDWAAQLQKYADSDELLREVAYWHNLETTTTTAALPTDFVTADRKQKHTRTLSFALTVPQTENLL
RHVHHAYHTEMNDLLLTALGLAVKDWAHTNGVVINLEGHGREDIQNEMNVTRTIGWFTSQYPVVLDMEKAEDL
PYQIKQTKENLRRIPKKGIGYEILRTLTTSQLQPPLAFTLRPEISFNYLGQFESDGKTGGFTFSPLGTGQLFSPESERVF
LLDISAMIEDGELRISVGYSRLQYEEKTIASLADSYRKHLLGIIEHCMAKEEGEYTPSDLGDEELSMEELENILEWIGS
RSHHHHHH

DNA sequences

pCTRB vector backbone

TAGGTCGAGATCTGATAACAACAGTGTAGATGTAACAAAATCGACTTTGTTCCCACTGTACTTTTAGCTCGTAC
AAAATACAATATACTTTTCATTTCTCCGTAAACAACATGTTTTCCCATGTAATATCCTTTTCTATTTTTCGTTCC
GTTACCAACTTTACACATACTTTATATAGCTATTCACTTCTATACACTAAAAAACTAAGACAATTTTAATTTTG
CTGCCTGCCATATTTCAATTTGTTATAAATTCCTATAATTTATCCTATTAGTAGCTAAAAAAAGATGAATGTGA
ATCGAATCCTAAGAGAATTGAGCTCCAATTCGCCCTATAGTGAGTCGTATTACAATTCACTGGCCGTCGTTTTA
CAACGTCGTGACTGGGAAAACCCTGGCGTTACCCAACTTAATCGCCTTGCAGCACATCCCCCCTTCGCCAGCT
GGCGTAATAGCGAAGAGGCCCGCACCGATCGCCCTTCCCAACAGTTGCGCAGCCTGAATGGCGAATGGCGCG
ACGCGCCCTGTAGCGGCGCATTAAGCGCGGCGGGTGTGGTGGTTACGCGCAGCGTGACCGCTACACTTGCCAG
CGCCCTAGCGCCCGCTCCTTTCGCTTTCTTCCCTTCCTTTCTCGCCACGTTCGCCGGCTTTCCCCGTCAAGCTCT
AAATCGGGGGCTCCCTTTAGGGTTCCGATTTAGTGCTTTACGGCACCTCGACCCCAAAAAACTTGATTAGGGT
GATGGTTCACGTAGTGGGCCATCGCCCTGATAGACGGTTTTTCGCCCTTTGACGTTGGAGTCCACGTTCTTTAA
TAGTGGACTCTTGTTCCAAACTGGAACAACACTCAACCCTATCTCGGTCTATTCTTTTGATTTATAAGGGATTT
TGCCGATTTCGGCCTATTGGTTAAAAAATGAGCTGATTTAACAAAAATTTAACGCGAATTTTAACAAAATATT
AACGTTTACAATTTCCTGATGCGGTATTTTCTCCTTACGCATCTGTGCGGTATTTCACACCGCAGGCAAGTGCA
CAAACAATACTTAAATAAATACTACTCAGTAATAACCTATTTCTTAGCATTTTTGACGAAATTTGCTATTTTGT
TAGAGTCTTTTACACCATTTGTCTCCACACCTCCGCTTACATCAACACCAATAACGCCATTTAATCTAAGCGCA
TCACCAACATTTTCTGGCGTCAGTCCACCAGCTAACATAAAATGTAAGCTTTCGGGGCTCTCTTGCCTTCCAAC
CCAGTCAGAAATCGAGTTCCAATCCAAAAGTTCACCTGTCCCACCTGCTTCTGAATCAAACAAGGGAATAAAC
GAATGAGGTTTCTGTGAAGCTGCACTGAGTAGTATGTTGCAGTCTTTTGGAAATACGAGTCTTTTAATAACTGG
CAAACCGAGGAACTCTTGGTATTCTTGCCACGACTCATCTCCATGCAGTTGGACGATATCAATGCCGTAATCAT
TGACCAGAGCCAAAACATCCTCCTTAGGTTGATTACGAAACACGCCAACCAAGTATTTCGGAGTGCCTGAACT
ATTTTTATATGCTTTTACAAGACTTGAAATTTTCCTTGCAATAACCGGGTCAATTGTTCTCTTTCTATTGGGCAC
ACATATAATACCCAGCAAGTCAGCATCGGAATCTAGAGCACATTCTGCGGCCTCTGTGCTCTGCAAGCCGCAA
ACTTTCACCAATGGACCAGAACTACCTGTGAAATTAATAACAGACATACTCCAAGCTGCCTTTGTGTGCTTAAT
CACGTATACTCACGTGCTCAATAGTCACCAATGCCCTCCCTCTTGGCCCTCTCCTTTTCTTTTTTCGACCGAATT
AATTCTTAATCGGCAAAAAAAGAAAAGCTCCGGATCAAGATTGTACGTAAGGTGACAAGCTATTTTTCAATAA
AGAATATCTTCCACTACTGCCATCTGGCGTCATAACTGCAAAGTACACATATATTACGATGCTGTCTATTAAAT
GCTTCCTATATTATATATATAGTAATGTCGTTTATGGTGCACTCTCAGTACAATCTGCTCTGATGCCGCATAGTT
AAGCCAGCCCCGACACCCGCCAACACCCGCTGACGCGCCCTGACGGGCTTGTCTGCTCCCGGCATCCGCTTAC
AGACAAGCTGTGACCGTCTCCGGGAGCTGCATGTGTCAGAGGTTTTCACCGTCATCACCGAAACGCGCGAGAC
GAAAGGGCCTCGTGATACGCCTATTTTTATAGGTTAATGTCATGATAATAATGGTTTCTTAGGACGGATCGCTT
GCCTGTAACTTACACGCGCCTCGTATCTTTTAATGATGGAATAATTTGGGAATTTACTCTGTGTTTATTTATTTT
TATGTTTTGTATTTGGATTTTAGAAAGTAAATAAAGAAGGTAGAAGAGTTACGGAATGAAGAAAAAAAAATA
AACAAAGGTTTAAAAAATTTCAACAAAAAGCGTACTTTACATATATATTTATTAGACAAGAAAAGCAGATTAA
ATAGATATACATTCGATTAACGATAAGTAAAATGTAAAATCACAGGATTTTCGTGTGTGGTCTTCTACACAGA
CAAGATGAAACAATTCGGCATTAATACCTGAGAGCAGGAAGAGCAAGATAAAAGGTAGTATTTGTTGGCGAT
CCCCCTAGAGTCTTTTACATCTTCGGAAAACAAAAACTATTTTTTCTTTAATTTCTTTTTTTACTTTCTATTTTTA
ATTTATATATTTATATTAAAAAATTTAAATTATAATTATTTTTATAGCACGTGATGAAAAGGACCCAGGTGGCA
CTTTTCGGGGAAATGTGCGCGGAACCCCTATTTGTTTATTTTTCTAAATACATTCAAATATGTATCCGCTCATG
AGACAATAACCCTGATAAATGCTTCAATAATATTGAAAAAGGAAGAGTATGAGTATTCAACATTTCCGTGTCG
CCCTTATTCCCTTTTTTGCGGCATTTTGCCTTCCTGTTTTTGCTCACCCAGAAACGCTGGTGAAAGTAAAAGATG
CTGAAGATCAGTTGGGTGCACGAGTGGGTTACATCGAACTGGATCTCAACAGCGGTAAGATCCTTGAGAGTTT
TCGCCCCGAAGAACGTTTTCCAATGATGAGCACTTTTAAAGTTCTGCTATGTGGCGCGGTATTATCCCGTATTG
ACGCCGGGCAAGAGCAACTCGGTCGCCGCATACACTATTCTCAGAATGACTTGGTTGAGTACTCACCAGTCAC
AGAAAAGCATCTTACGGATGGCATGACAGTAAGAGAATTATGCAGTGCTGCCATAACCATGAGTGATAACAC
TGCGGCCAACTTACTTCTGACAACGATCGGAGGACCGAAGGAGCTAACCGCTTTTTTTCACAACATGGGGGAT
CATGTAACTCGCCTTGATCGTTGGGAACCGGAGCTGAATGAAGCCATACCAAACGACGAGCGTGACACCACG
ATGCCTGTAGCAATGGCAACAACGTTGCGCAAACTATTAACTGGCGAACTACTTACTCTAGCTTCCCGGCAAC
AATTAATAGACTGGATGGAGGCGGATAAAGTTGCAGGACCACTTCTGCGCTCGGCCCTTCCGGCTGGCTGGTT
TATTGCTGATAAATCTGGAGCCGGTGAGCGTGGGTCTCGCGGTATCATTGCAGCACTGGGGCCAGATGGTAAG
CCCTCCCGTATCGTAGTTATCTACACGACGGGCAGTCAGGCAACTATGGATGAACGAAATAGACAGATCGCTG
AGATAGGTGCCTCACTGATTAAGCATTGGTAACTGTCAGACCAAGTTTACTCATATATACTTTAGATTGATTTA
AAACTTCATTTTTAATTTAAAAGGATCTAGGTGAAGATCCTTTTTGATAATCTCATGACCAAAATCCCTTAACG
TGAGTTTTCGTTCCACTGAGCGTCAGACCCCGTAGAAAAGATCAAAGGATCTTCTTGAGATCCTTTTTTTCTGC
GCGTAATCTGCTGCTTGCAAACAAAAAAACCACCGCTACCAGCGGTGGTTTGTTTGCCGGATCAAGAGCTACC
AACTCTTTTTCCGAAGGTAACTGGCTTCAGCAGAGCGCAGATACCAAATACTGTCCTTCTAGTGTAGCCGTAGT
TAGGCCACCACTTCAAGAACTCTGTAGCACCGCCTACATACCTCGCTCTGCTAATCCTGTTACCAGTGGCTGCT
GCCAGTGGCGATAAGTCGTGTCTTACCGGGTTGGACTCAAGACGATAGTTACCGGATAAGGCGCAGCGGTCG
GGCTGAACGGGGGGTTCGTGCACACAGCCCAGCTTGGAGCGAACGACCTACACCGAACTGAGATACCTACAG
CGTGAGCATTGAGAAAGCGCCACGCTTCCCGAAGGGAGAAAGGCGGACAGGTATCCGGTAAGCGGCAGGGTC
GGAACAGGAGAGCGCACGAGGGAGCTTCCAGGGGGAAACGCCTGGTATCTTTATAGTCCTGTCGGGTTTCGCC
ACCTCTGACTTGAGCGTCGATTTTTGTGATGCTCGTCAGGGGGGCCGAGCCTATGGAAAAACGCCAGCAACGC
GGCCTTTTTACGGTTCCTGGCCTTTTGCTGGCCTTTTGCTCACATGTTCTTTCCTGCGTTATCCCCTGATTCTGTG

GATAACCGTATTACCGCCTTTGAGTGAGCTGATACCGCTCGCCGCAGCCGAACGACCGAGCGCAGCGAGTCA
GTGAGCGAGGAAGCGGAAGAGCGCCCAATACGCAAACCGCCTCTCCCCGCGCGTTGGCCGATTCATTAATGC
AGCTGGCACGACAGGTTTCCCGACTGGAAAGCGGGCAGTGAGCGCAACGCAATTAATGTGAGTTACCTCACT
CATTAGGCACCCCAGGCTTTACACTTTATGCTTCCGGCTCCTATGTTGTGTGGAATTGTGAGCGGATAACAATT
TCACACAGGAAACAGCTATGACCATGATTACGCCAAGCTCGGAATTAACCCTCACTAAAGGGAACAAAAGCT
GGGTACCCGACAGGTTATCAGCAACAACACAGTCATATCCATTCTCAATTAGCTCTACCACAGTGTGTGAACC
AATGTATCCAGCACCACCTGTAACCAAAACAATTTTAGAAGTACTTTCACTTTGTAACTGAGCTGTCATTTATA
TTGAATTTTCAAAAATTCTTACTTTTTTTTTGGATGGACGCAAAGAAGTTTAATAATCATATTACATGGCATTA
CCACCATATACATATCCATATACATATCCATATCTAATCTTACTTATATGTTGTGGAAATGTAAAGAGCCCCAT
TATCTTAGCCTAAAAAAACCTTCTCTTTGGAACTTTCAGTAATACGCTTAACTGCTCATTGCTATATTGAAGTA
CGGATTAGAAGCCGCCGAGCGGGTGACAGCCCTCCGAAGGAAGACTCTCCTCCGTGCGTCCTCGTCTTCACCG
GTCGCGTTCCTGAAACGCAGATGTGCCTCGCGCCGCACTGCTCCGAACAATAAAGATTCTACAATACTAGCTT
TTATGGTTATGAAGAGGAAAAATTGGCAGTAACCTGGCCCCACAAACCTTCAAATGAACGAATCAAATTAAC
AACCATAGGATGATAATGCGATTAGTTTTTTAGCCTTATTTCTGGGGTAATTAATCAGCGAAGCGATGATTTTT
GATCTATTAACAGATATATAAATGCAAAAACTGCATAACCACTTTAACTAATACTTTCAACATTTTCGGTTTGT
ATTACTTCTTATTCAAATGTAATAAAAGATCGAATTCTACTTCATACATTTTCAATTAAG (Open reading frame)

Aga-HA-W2742S-TycB3-A-T-c-myc (ORF of pCTRB-W2742S-TycB3 A-T with the start codon in bold)

ATGCAGTTACTTCGCTGTTTTTCAATATTTTCTGTTATTGCTTCAGTTTTAGCACAGGAACTGACAACTATATGC
GAGCAAATCCCCTCACCAACTTTAGAATCGACGCCGTACTCTTTGTCAACGACTACTATTTTGGCCAACGGGA
AGGCAATGCAAGGAGTTTTTGAATATTACAAATCAGTAACGTTTGTCAGTAATTGCGGTTCTCACCCCTCAAC
AACTAGCAAAGGCAGCCCCATAAACACACAGTATGTTTTTAAGGACAATAGCTCGACGATTGAAGGTAGATA
CCCATACGACGTTCCAGACTACGCTCTGCAGGCTAGTGGTGGTGGTGGTTCTGGTGGTGGTGGTTCTGGTGGT
GGTGGTTCTCAT**ATG**TTCAACGATACGCACAGAGAATACCGGGCAGATCAAACAATCCAGCAACTTTTTGAAG
AGCTGGCGGAAAAAATGCCTGAGCACACGGCGCTCGTATTCGAAGAAAAGCGCATGTCGTTCCGGGAGCTCA
ATGAAAGAGCGAACCAGCTCGCAGCCGTTTTGCGGGAAAAAGGAGTCGGGCCAGCGCAGATCGTCGCTTTGC
TGGTAGAGCGTTCCGCCGAGATGGTCATTGCCACGCTTGCCACGTTAAAAGCGGGCGGCGCCTTTTTGCCCGT
CGATCCTGATTATCCGGAAGAGCGAATCCGCTACATGCTGGAGGACAGCCAGGCAAAACTGGTGGTGACCCA
TGCGCACTTGCTGCACAAAGTGAGCAGTCAGTCCGAAGTCGTTGATGTGGATGACCCTGGAAGCTACGCAACA
CAGACAGACAACCTGCCGTGCGCAAACACACCGTCTGATTTGGCTTATATCATTTACACGTCCGGTACGACGG
GCAAGCCAAAAGGCGTCATGCTGGAGCACAAAGGGGTAGCGAATCTGCAAGCGGTATTTGCCCATCATCTAG
GCGTCACGCCGCAAGATCGGGCAGGGCATTTTGCCAGCATCTCGTTTGACGCATCGGTGTCGGATATGTTTGG
CCCGTTGCTGTCGGGAGCGACCTTGTACGTCTTGTCCCGAGACGTCATCAACGATTTTCAACGATTCGCCGAAT
ACGTTCGCGATAACGCGATCACCTTCCTCACTTTGCCGCCGACGTACGCGATTTATCTGGAGCCGGAGCAGGT
GCCGTCGTTACGCACCCTGATTACAGCCGGATCGGCTTCCTCCGTTGCATTGGTGGATAAATGGAAAGAAAAA
GTCACCTATGTCAATGGATACGGCCCAACAGAGAGCACCGTTTGCGCGACACTGTGGAAAGCCAAACCGGAT
GAGCCAGTCGAAACGATCACGATTGGCAAACCGATTCAGAACACCAAGCTGTACATCGTGGATGACCAGTTG
CAGTTGAAAGCGCCGGGGCAGATGGGAGAACTGTGCATCAGCGGCTTGTCGCTGGCGAGAGGCTATTGGAAT
CGTCCAGAGCTGACCGCCGAGAAGTTCGTCGACAACCCGTTTGTGCCAGGAACAAAGATGTACCGGACAGGC
GACCTGGCAAGATGGCTGCCAGATGGAACTATCGAGTATCTGGGCAGAATCGATCACCAAGTGAAAATTCGC
GGACATCGTGTGGAACTCGGCGAAGTGGAAAGCGTGCTGCTGCGGTATGACACGGTCAAAGAGGCGGCCGCC
ATCACACATGAGGACGACCGCGGCCAAGCTTACTTGTGCGCCTACTACGTAGCGGAGGGAGAAGCCACGCCT
GCGCAACTTCGAGCCTATATGGAAAACGAGTTGCCGAACTACATGGTTCCCGCCTTCTTTATCCAGTTGGAAA
AGATGCCGCTGACACCGAATGACAAGATTGACCGAAAAGCGCTGCCGAAGCCGAACCAGGAGGAGAACCGG
ACTGAGCAATATGCAGCGCCGCAAACCGAGCTGGAACAGTTGCTGGCTGGCATCTGGGCAGATGTACTGGGG
ATCAAGCAAGTCGGGACGCAAGACAACTTCTTTGAATTGGGCGGCGATTCGATTAAAGCGATCCAGGTATCCA
CCCGCCTGAATGCGTCAGGCTGGACGCTTGCGATGAAAGAACTGTTCCAGTACCCGACGATTGAAGAAGCTGC
TCTGCGCGTCATCCCGAACAGCCGAGAGAGCGAGCAGGGTGTCCTCGAG**GAACAAAAGCTTATTTCTGAAG
AGGACTTG**

Aga-HA-W2742S-TycB3-C-A-T-c-myc (ORF of pCTRB-W2742S-TycB3 C-A-T with the start codon in bold)

ATGCAGTTACTTCGCTGTTTTTCAATATTTTCTGTTATTGCTTCAGTTTTAGCACAGGAACTGACAACTATATGC
GAGCAAATCCCCTCACCAACTTTAGAATCGACGCCGTACTCTTTGTCAACGACTACTATTTTGGCCAACGGGA
AGGCAATGCAAGGAGTTTTTGAATATTACAAATCAGTAACGTTTGTCAGTAATTGCGGTTCTCACCCCTCAAC
AACTAGCAAAGGCAGCCCCATAAACACACAGTATGTTTTTAAGGACAATAGCTCGACGATTGAAGGTAGATA
CCCATACGACGTTCCAGACTACGCTCTGCAGGCTAGTGGTGGTGGTGGTTCTGGTGGTGGTGGTTCTGGTGGT
GGTGGTTCTCAT**ATG**GAACAGGCAGCCGGCGCCGCCATTCAACCGGCAGAGCCGCAAGCGTATTATCCGGTA
TCTTCTGCTCAGCAGCGGATGTACTTGCTCCATCAGCTTGAAGGTGCCGGAATCAGCTACAACACACCGGGCA
TCATCATGCTGGAAGGCAAGCTCGATCGCGAGCAATTGGCGAATGCGCTGCAAGCGTTGGTAGATCGTCACGA
TATTTTGCGGACGTCTTTTGAGATGGTCGGAGACGAGCTGGTGCAAAAAATTCATGACCGCGTGGCCGTGAAC
ATGGAGTATGTGACGGCAGAAGAGCAGCAGATCGATGACCTTTTCCACGCGTTCGTCCGTCCGTTTGATCTTTC
TGTGCCGCCATTGCTCCGCATGAGCCTGGTGAAACTCGCGGATGAGCGTCACCTGCTCCTGTACGACATGCAC

CATATTGCTGCCGATGCCGCATCGATCACGATCCTGTTCGATGAACTGGCTGAATTGTACCAGGGAAGAGAAC
TGCCGGAAATGCGCATCCAGTACAAAGATTTTGCTGTCTGGCAAAAAGCCTTGCATGAGTCGGATGCCTTCAA
GCAGCAGGAAGCGTATTGGCTGAGCACGTTCGCTGGAAATATCACCGCTGTCGATGTGCCGACAGATTTTCCG
CGCCCAGCCGTGAAAAGTTTTGCAGGGGGGCAAGTCACCCTGTCCATGGACCAAGAGCTGCTCAGTGCTTTGC
ACGAGTTGGCTGCGCATACGAATACGACGCTGTTTATGGTTTTGCTGGCCGCCTACAACGTGCTGCTCGCAAA
ATACGCTGGGCAAGACGACATCATCGTGGGAACGCCGATCTCCGGCAGGTCACGCGCCGAGCTTGCGCCTGTC
GTCGGCATGTTCGTCCATACGCTGGCGATCCGCAACAAACCGACCGCCGAGAAGACATTCAAGCAGTTTTTGC
AGGAGGTCAAGCAAAACGCGCTCGATGCTTTCGACCACCAGGACTACCCGTTTGAAAGCCTTGTGGAAAAGC
TGGGCATTCCGCGCGATCCGGGGCGCAATCCGCTGTTTGACACCATGTTCATCCTGCAAAACGATGAGTTGCA
CGCAAAAACGCTGGATCAGCTCGTCTATCGCCCTTATGAATCGGACAGCGCGCTTGACGTGGCGAAATTCGAC
TTGTCGTTCCATCTGACCGAGCGGGAAACCGACCTGTTCTTGCGCCTGGAATACTGCACCAAGCTGTTCAAGC
AACAAACGGTAGAACGAATGGCGCACCACTTCTTGCAAATTTTGCGAGCGGTCACGGCCAATCCGGAAAATG
AATTGCAAGAGATCGAGATGCTGACAGCAGCAGAAAAGCAAATGCTGCTGGTGGCGTTCAACGATACGCACA
GAGAATACCGGGCAGATCAAACAATCCAGCAACTTTTTGAAGAGCTGGCGGAAAAAATGCCTGAGCACACGG
CGCTCGTATTCGAAGAAAAGCGCATGTCGTTCCGGGAGCTCAATGAAAGAGCGAACCAGCTCGCAGCCGTTTT
GCGGGAAAAAGGAGTCGGGCCAGCGCAGATCGTCGCTTTGCTGGTAGAGCGTTCCGCCGAGATGGTCATTGC
CACGCTTGCCACGTTAAAAGCGGGCGGCGCCTTTTTGCCCGTCGATCCTGATTATCCGGAAGAGCGAATCCGC
TACATGCTGGAGGACAGCCAGGCAAAACTGGTGGTGACCCATGCGCACTTGCTGCACAAAGTGAGCAGTCAG
TCCGAAGTCGTTGATGTGGATGACCCTGGAAGCTACGCAACACAGACAGACAACCTGCCGTGCGCAAACACA
CCGTCTGATTTGGCTTATATCATTTACACGTCCGGTACGACGGGCAAGCCAAAAGGCGTCATGCTGGAGCACA
AAGGGGTAGCGAATCTGCAAGCGGTATTTGCCCATCATCTAGGCGTCACGCCGCAAGATCGGGCAGGGCATTT
TGCCAGCATCTCGTTTGACGCATCGGTGTCGGATATGTTTGGCCCGTTGCTGTCGGGAGCGACCTTGTACGTCT
TGTCCCGAGACGTCATCAACGATTTTCAACGATTCGCCGAATACGTTCGCGATAACGCGATCACCTTCCTCACT
TTGCCGCCGACGTACGCGATTTATCTGGAGCCGGAGCAGGTGCCGTCGTTACGCACCCTGATTACAGCCGGAT
CGGCTTCCTCCGTTGCATTGGTGGATAAATGGAAAGAAAAAGTCACCTATGTCAATGGATACGGCCCAACAGA
GAGCACCGTTTGCGCGACACTGTGGAAAGCCAAACCGGATGAGCCAGTCGAAACGATCACGATTGGCAAACC
GATTCAGAACACCAAGCTGTACATCGTGGATGACCAGTTGCAGTTGAAAGCGCCGGGGCAGATGGGAGAACT
GTGCATCAGCGGCTTGTCGCTGGCGAGAGGCTATTGGAATCGTCCAGAGCTGACCGCCGAGAAGTTCGTCGAC
AACCCGTTTGTGCCAGGAACAAAGATGTACCGGACAGGCGACCTGGCAAGATGGCTGCCAGATGGAACTATC
GAGTATCTGGGCAGAATCGATCACCAAGTGAAAATTCGCGGACATCGTGTGGAACTCGGCGAAGTGGAAAGC
GTGCTGCTGCGGTATGACACGGTCAAAGAGGCGGCCGCCATCACACATGAGGACGACCGCGGCCAAGCTTAC
TTGTGCGCCTACTACGTAGCGGAGGGAGAAGCCACGCCTGCGCAACTTCGAGCCTATATGGAAAACGAGTTG
CCGAACTACATGGTTCCCGCCTTCTTTATCCAGTTGGAAAAGATGCCGCTGACACCGAATGACAAGATTGACC
GAAAAGCGCTGCCGAAGCCGAACCAGGAGGAGAACCGGACTGAGCAATATGCAGCGCCGCAAACCGAGCTG
GAACAGTTGCTGGCTGGCATCTGGGCAGATGTACTGGGGATCAAGCAAGTCGGGACGCAAGACAACTTCTTTG
AATTGGGCGGCGATTCGATTAAAGCGATCCAGGTATCCACCCGCCTGAATGCGTCAGGCTGGACGCTTGCGAT
GAAAGAACTGTTCCAGTACCCGACGATTGAAGAAGCTGCTCTGCGCGTCATCCCGAACAGCCGAGAGAGCGA
GCAGGGTGTCCTCGAG**GAACAAAAGCTTATTTCTGAAGAGGACTTG**

Aga-HA-TycC3-A-T-c-myc (ORF of pCTRB-TycC3 A-T with the start codon in bold)

ATGCAGTTACTTCGCTGTTTTTCAATATTTTCTGTTATTGCTTCAGTTTTAGCACAGGAACTGACAACTATATGC
GAGCAAATCCCCTCACCAACTTTAGAATCGACGCCGTACTCTTTGTCAACGACTACTATTTTGGCCAACGGGA
AGGCAATGCAAGGAGTTTTTGAATATTACAAATCAGTAACGTTTGTCAGTAATTGCGGTTCTCACCCCTCAAC
AACTAGCAAAGGCAGCCCCATAAACACACAGTATGTTTTTAAGGACAATAGCTCGACGATTGAAGGTAGATA
CCCATACGACGTTCCAGACTACGCTCTGCAGGCTAGTGGTGGTGGTGGTTCTGGTGGTGGTGGTTCTGGTGGT
GGTGGTTCTCAT**ATG**TTCAACGGTGCGCACAAAGATTTTCCGCAAAACAAAACGCTTCAGGCGCTTTTTGAAG
AACAAGCGGAAAAGTCGCCGCAGGCAACAGCCGTGGAAATCAGCGGGCAGCCCCTGTCCTATCAGGAGCTGA
ATGAGCGAGCCAACCAGCTTGCCGCTACGCTGCGGGAGCGGGGAGTACAGCCTGACCAACCTGTAGGGATTA
TGGCGAACCGCTCTGTGGAGATGGTCGTCGGCATCCTCGCCATCTTGAAAGCAGGCGGAGCTTACGTGCCGAT
CGACCCGGAATATCCGGAGGAGCGTGTCGCCTACATGCTGACGGATTGCCAAGCCCGCCTGGTGCTGACGCA
AAAGCATCTGGGAGCGAAGCTTGGTTCCAGCGTGACCGCGGAATGCCTGTATCTCGACGACGAGAGCAACTA
TGGTGTGCACCGCTCGAATTTGCAGCCGATCAATACCGCTTCCGATCTGGCTTACATCATCTACACATCGGGTA
CGACTGGCAAGCCAAAAGGGGTCATGGTCGAGCACCGGGGCATCGTCAACAACGTGCTGTGGAAGAAAGCGG
AGTACCAAATGAAGGTTGGCGACAGAAGCTTGCTGTCTCTGTCCTTTGCCTTTGACGCTTTCGTTCTGTCCTTCT
TTACGCCTGTGCTTTCCGGGGCAACTGTCGTACTGGCGGAGGATGAAGAAGCCAAGGACCCAGTCTCTTTGAA
AAAGCTCATCGCCGCTTCGCGCTGCACCTTGATGACAGGCGTGCCGAGCTTGTTCCAGGCCATTCTGGAATGC
AGCACGCCAGCGGATATCCGTCCGCTGCAAACCGTCACACTCGGCGGAGAAAAAATTACGGCGCAGCTTGTT
GAAAAATGCAAGCAGCTGAATCCCGATCTGGTCATCGTCAACGAGTACGGCCCGACAGAAAGCAGTGTCGTC
GCCACCTGGCAGCGCCTTGCGGGTCCGGATGCTGCCATCACCATCGGGCGGCCGATTGCCAACACCAGCCTGT
ACATCGTGAACCAATATCACCAGCTACAGCCAATCGGCGTGGTCGGGGAGATTTGCATCGGCGGCCGCGGCTT
GGCACGAGGCTATTGGAACAAGCCAGCGCTCACGGAAGAGAAGTTCGTTTCCCATCCGTTTGCGGCAGGCGA
GCGCATGTACAAGACGGGCGATCTTGGCAAGTGGCTCCCGGACGGAACGATTGAATACATTGGGCGCATCGA
CGAACAGGTCAAAGTCCGAGGCTACCGAATTGAAATCGGCGAGATCGAGTCGGCTCTGCTGGCTGCGGAAAA
GCTGACAGCGGCTGTTGTGGTCGTCTATGAGGATCAGCTTGGCCAGTCGGCTCTGGCAGCGTATTTACCGCC
GACGAACAGCTTGATGTCACGAAGCTGTGGTCGCATCTGTCGAAGCGACTCCCGTCGTACATGATTCCTGCGC

ATTTTGTGCAGCTCGATCAGCTTCCGCTTACGCCAAACGGCAAAGTCGACAAGAAAGCCTTGCCGAAGCCAGA
AGGCAAGCCCGTAACCGAAGCGCAATATGTCGCGCCGACAAATGCGGTGGAAAGCAAGCTGGCAGAGATTTG
GGAACGCGTGCTCGGGGTTAGCGGCATCGGCATTCTCGACAACTTTTTCCAGATCGGCGGACATTCCTTGAAA
GCGATGGCTGTCGCTGCACAGGTGCATCGCGAGTATCAGGTTGAGCTTCCGCTGAAAGTGCTGTTCGCGCAGC
CTACGATCAAGGCGTTGGCCCAGTATGTCGCCACGAGCGGAAAAGAGACGTATGTGCCGATCCTCGAG**GAAC
AAAAGCTTATTTCTGAAGAGGACTTG**

Aga-HA-TycC6-C-A-T-c-myc (ORF of pCTRB-TycC6 C-A-T with the start codon in bold)

ATGCAGTTACTTCGCTGTTTTTCAATATTTTCTGTTATTGCTTCAGTTTTAGCACAGGAACTGACAACTATATGC
GAGCAAATCCCCTCACCAACTTTAGAATCGACGCCGTACTCTTTGTCAACGACTACTATTTTGGCCAACGGGA
AGGCAATGCAAGGAGTTTTTGAATATTACAAATCAGTAACGTTTGTCAGTAATTGCGGTTCTCACCCCTCAAC
AACTAGCAAAGGCAGCCCCATAAACACACAGTATGTTTTAAGGACAATAGCTCGACGATTGAAGGTAGATA
CCCATACGACGTTCCAGACTACGCTCTGCAGGCTAGTGGTGGTGGTGGTTCTGGTGGTGGTGGTTCTGGTGGT
GGTGGTTCTCAT**ATG**GCCAAAGGGAATGTCTTCTCGATCGAGCCTGTGCAAAAGCAAGCGTACTATCCGGTCT
CCTCGGCACAAAAGCGCATGTACATCCTCGATCAATTTGAGGGAGTCGGCATCAGCTACAACATGCCGTCGAC
TATGCTGATCGAAGGCAAGCTGGAGCGAACACGGGTAGAAGCGGCGTTCCAGCGCTTGATTGCGCGACATGA
AAGCCTGCGCACTTCGTTTGCCGTCGTCAACGGAGAGCCTGTGCAAAACATTCACGAGGACGTTCCGTTTGCG
CTTGCCTATTCGGAAGTCACAGAACAGGAGGCGCGCGAACTCGTTTCTTCTCTCGTGCAGCCGTTCGATCTGG
AGGTCGCACCACTCATCCGCGTGTCGCTGCTGAAAATCGGCGAGGATCGTTACGTGCTCTTTACCGACATGCA
TCACAGCATTTCCGATGGCGTATCCTCCGGCATTCTTTTGGCAGAGTGGGTGCAGCTGTACCAGGGTGACGTTT
TGCCGGAGCTGCGTATCCAGTACAAGGACTTTGCTGTGTGGCAACAAGAGTTTTCCCAGTCGGCTGCCTTCCA
CAAGCAGGAAGCGTACTGGTTGCAAACGTTTGCCGATGACATTCCTGTGCTGAACTTGCCGACCGATTTCACC
CGCCCCAGCACCCAAAGCTTTGCCGGGGATCAGTGCACGATCGGCGCGGGCAAAGCGCTCACGGAAGGCTTG
CACCAGTTGGCGCAGGCGACGGGAACGACTTTGTACATGGTTTTGCTCGCCGCGTACAACGTGCTGCTCGCCA
AGTATGCCGGGCAGGAGGACATCATCGTCGGCACGCCGATTACAGGCAGATCCCATGCCGATCTCGAACCGA
TCGTCGGCATGTTCGTGAACACCTTGGCGATGCGAAACAAACCGCAGCGCGAAAAGACTTTTAGCGAGTTTTT
GCAAGAAGTCAAGCAAAATGCGCTGGATGCGTACGGCCATCAGGATTACCCGTTTGAAGAACTGGTGGAAAA
GCTCGCGATCGCGCGCGATTTGAGCCGAAATCCGCTGTTTGACACCGTGTTTACGTTCCAAAACAGCACGGAA
GAGGTCATGACGCTGCCTGAATGCACGCTTGCGCCGTTTATGACGGACGAAACAGGCCAGCACGCCAAGTTC
GACTTGACTTTCAGCGCTACGGAAGAGCGGGAAGAAATGACGATTGGCGTGGAGTACAGCACAAGCTTGTTT
ACGCGGGAAACGATGGAACGGTTCAGCCGCCACTTCCTGACGATTGCAGCGAGCATCGTGCAAAATCCGCAC
ATCCGTCTGGGCGAGATCGACATGCTTTTGCCAGAAGAAAAACAGCAGATTTTGGCCGGGTTCAACGATACGG
CAGTCAGCTATGCGCTGGACAAAACGCTGCACCAGCTATTCGAAGAGCAGGTCGACAAAACACCGGATCAGG
CAGCGCTTCTCTTTAGCGAGCAATCGCTGACGTACAGCGAACTGAACGAGCGAGCAAACAGACTGGCAAGGG
TCCTGCGCGCAAAAGGAGTCGGACCGGACCGTCTGGTAGCGATCATGGCGGAGCGCTCGCCGGAAATGGTGA
TCGGTATTCTCGGTATTTTGAAGGCAGGCGGCGCTTATGTTCCCGTCGATCCCGGCTATCCGCAGGAGCGCATT
CAGTACCTGCTCGAAGATAGCAACGCAGCCCTGCTGCTCAGCCAGGCGCATCTGTTGCCGCTGTTGGCCCAGG
TGTCAAGCGAGCTGCCGGAGTGCCTTGATCTGAACGCTGAACTGGATGCCGGACTGAGCGGCTCCAACCTGCC
AGCTGTCAACCAACCGACTGACCTTGCCTACGTCATCTATACATCCGGTACGACCGGCAAGCCGAAGGGTGTC
ATGATCCCGCATCAAGGAATCGTGAACTGCTTGCAGTGGAGAAGAGACGAATACGGGTTCGGGCCGAGTGAC
AAGGCGTTGCAAGTGTTCTCCTTTGCCTTCGACGGTTTTGTAGCCAGCTTGTTCGCTCCGCTGCTCGGAGGGGC
AACGTGCGTGTTGCCGCAAGAAGCAGCTGCCAAAGACCCGGTCGCGCTGAAAAAACTGATGGCCGCAACGGA
AGTCACCCATTACTACGGCGTACCGAGTCTGTTCCAGGCCATTCTCGATTGCTCGACGACAACCGACTTCAATC
AGTTGCGTTGCGTCACTTTGGGCGGCGAGAAGCTGCCTGTGCAGCTTGTGCAAAAAACAAAAGAAAAGCATC
CGGCAATCGAGATCAACAACGAGTACGGCCCGACGGAAAACAGCGTCGTCACCACCATCTCGCGCTCGATTG
AAGCGGGGCAAGCGATCACGATTGGCCGACCGCTTGCGAACGTCCAAGTCTACATTGTAGATGAGCAGCATC
ACTTGCAGCCGATTGGCGTGGTCGGTGAGCTGTGCATCGGCGGAGCCGGGCTTGCCAGAGGCTATCTGAACAA
ACCGGAGCTGACCGCAGAGAAGTTTGTCGCAAATCCGTTCCGACCAGGCGAGCGCATGTACAAAACAGGCGA
CTTGGTAAAATGGCGGACGGATGGCACGATCGAGTACATCGGCCGCGCAGACGAACAGGTCAAGGTGAGAGG
GTATCGCATCGAGATCGGCGAGATCGAGAGCGCCGTACTCGCTTACCAGGGCATCGATCAAGCGGTGGTCGTT
GCGCGAGACGATGACGCTACGGCTGGTTCCTATCTTTGCGCCTACTTTGTCGCAGCAACAGCCGTGTCCGTATC
CGGCTTGAGAAGCCATCTGGCCAAAGAGCTGCCTGCTTACATGATTCCGAGCTATTTCGTCGAGCTGGATCAG
CTGCCGCTTTCCGCCAATGGAAAAGTGGATCGCAAAGCTTTGCCGAAGCCGCAACAGTCCGATGCGACCACGC
GCGAATACGTGGCCCCGAGGAATGCGACCGAACAGCAACTGGCAGCCATCTGGCAAGAAGTTTTGGGAGTAG
AGCCAATCGGCATCACCGACCAGTTCTTTGAACTCGGAGGACATTCCTTAAAAGCTACGCTGTTGATTGCCAA
AGTGTATGAGTACATGCAAATCGAGCTGCCGCTGAATCTCATCTTCCAGTATCCGACGATCGAAAAGGTGGCC
GATTTCATCACGCATAAGCGCTTTGAGAGCAGATACGGCACAGCCATTTTGTTACTCGAG**GAACAAAAGCTT
ATTTCTGAAGAGGACTTG**

Aga-HA-TycB2-A-T-c-myc (ORF of pCTRB-TycB2 A-T with the start codon in bold)

ATGCAGTTACTTCGCTGTTTTTCAATATTTTCTGTTATTGCTTCAGTTTTAGCACAGGAACTGACAACTATATGC
GAGCAAATCCCCTCACCAACTTTAGAATCGACGCCGTACTCTTTGTCAACGACTACTATTTTGGCCAACGGGA
AGGCAATGCAAGGAGTTTTTGAATATTACAAATCAGTAACGTTTGTCAGTAATTGCGGTTCTCACCCCTCAAC

AACTAGCAAAGGCAGCCCCATAAACACACAGTATGTTTTTAAGGACAATAGCTCGACGATTGAAGGTAGATA
CCCATACGACGTTCCAGACTACGCTCTGCAGGCTAGTGGTGGTGGTGGTTCTGGTGGTGGTGGTTCTGGTGGT
GGTGGTTCTCAT**ATG**CACTTCAACGACACGGCCGCCCTGTATCCAGCGGAGAGCACGCTGTCGCAGCTGTTTG
AAGATCAGGCACAGAAAACTCCTGAGCAAACCGCCGTCGTCTTCGGTGACAAACGACTGACGTACCGCGAAC
TGAACGAGCGGGCCAACCAGCTCGCGCACACTTTGCGGGCAAAAGGCGTGCAGGCTGAGCAAAGCGTAGGGA
TCATGGCGCAAAGATCGTTAGAGATGGCGATCGGAATCATCGCTATTCTCAAAGCGGGCGGGGCGTATGTGCC
GATCGATCCGGATTATCCGAATGAGCGGATTGCTTACATGCTGGAAGATTGCGAAGCCCGTCTGGTGCTGACC
CAGCAGCAGCTCGCCGAAAAGATGACCGCAAACGTGGAATGCCTGTATCTGGATGAGGAGGGCAGCTACTCG
CCTCAGACGGAAAACATCGAGCCGATCCATACCGCTGCTGATCTCGCTTACATCATCTACACATCCGGTACGA
CAGGCAGGCCAAAAGGCGTCATGGTAGAGCATCGGGGAATCGTCAACAGTGTGACGTGGAACAGGGACGAGT
TTGCCCTTTCTGTCCGGGACAGTGGAACGCTGTCGCTATCTTTTGCCTTCGATGCCTTTGCCCTTACTTTCTTTA
CGTTGATTGTATCAGGCTCCACGGTCGTCCTGATGCCGGATCACGAAGCCAAAGATCCGATCGCGCTACGCAA
CCTGATTGCCGCTTGGGAATGCAGCTACGTCGTTTTCGTGCCCAGTATGTTCCAGGCGATATTGGAGTGCAGCA
CTCCGGCAGACATCCGCTCCATCCAGGCAGTCATGCTCGGGGGCGAAAAGCTGTCGCCGAAGCTTGTTCAGCT
GTGCAAAGCGATGCATCCGCAGATGAGCGTGATGAATGCATACGGCCCGACGGAGAGCAGCGTCATGGCCAC
CTACCTGCGAGATACACAGCCAGATCAGCCGATCACCATCGGGCGGCCGATTGCCAACACCGCCATTTACATC
GTAGACCAGCACCATCAACTGCTGCCTGTCGGGGTGGTAGGGGAAATCTGCATCGGCGGTCACGGCTTGGCGC
GGGGCTATTGGAAAAAGCCGGAGCTTACTGCGGAGAAATTCGTGGCCAATCCAGCTGTTCCGGGAGAGCGCA
TGTACAAAACAGGCGATCTGGGCAGATGGCTCCACGACGGCACGATTGATTTTATAGGCCGCGTCGATGACCA
AATCAAGGTGAGAGGATACCGGATTGAGGTCGGGGAGATTGAAGCGGTTTTGCTCGCTTACGATCAGACGAA
TGAAGCTATCGTCGTCGCTTATCAGGACGATCGCGGCGATTCCTATCTGGCTGCGTATGTCACGGAAAAACG
GCGATAGAGGAATCCGAGCTTCGCGCGCATCTGTTGCGAGAGCTTCCGGCCTACATGGTGCCGACCTACCTGA
TTCAACTGGACGCTTTCCCGCTCACGCCAAACGGCAAGGTCGACCGCAAGGCACTGCCCAAGCCGGAAGGAA
AGCCTGCAACAGGAGCAGCTTATGTCGCACCCGCTACAGAAGTGGAGGCGAAGCTGGTCGCCATTTGGGAGA
ATGCGCTGGGGATTTCCGGCGTCGGGGTGTTGGATCACTTTTTTGAGCTGGGCGGTCATTCCTTGAAAGCGATG
ACGGTTGTGGCGCAAGTGCATCGCGAGTTTCAAATCGACCTTTTGCTGAAGCAGTTTTTTGCAGCGCCAACCAT
CCGGGACTTGGCCCGCTTGATCGAACATAGCGAACAGGCAGCCGGCGCCGCCATTCTCGAG**GAACAAAAGCT**
TATTTCTGAAGAGGACTTG

Aga-HA-M1768A/M1803I-TycB2-A-T-c-myc (ORF of pCTRB-M1768A/M1803I-TycB2 A-T with the start codon in bold)

ATGCAGTTACTTCGCTGTTTTTCAATATTTTCTGTTATTGCTTCAGTTTTAGCACAGGAACTGACAACTATATGC
GAGCAAATCCCCTCACCAACTTTAGAATCGACGCCGTACTCTTTGTCAACGACTACTATTTTGGCCAACGGGA
AGGCAATGCAAGGAGTTTTTGAATATTACAAATCAGTAACGTTTGTCAGTAATTGCGGTTCTCACCCCTCAAC
AACTAGCAAAGGCAGCCCCATAAACACACAGTATGTTTTTAAGGACAATAGCTCGACGATTGAAGGTAGATA
CCCATACGACGTTCCAGACTACGCTCTGCAGGCTAGTGGTGGTGGTGGTTCTGGTGGTGGTGGTTCTGGTGGT
GGTGGTTCTCAT**ATG**CACTTCAACGACACGGCCGCCCTGTATCCAGCGGAGAGCACGCTGTCGCAGCTGTTTG
AAGATCAGGCACAGAAAACTCCTGAGCAAACCGCCGTCGTCTTCGGTGACAAACGACTGACGTACCGCGAAC
TGAACGAGCGGGCCAACCAGCTCGCGCACACTTTGCGGGCAAAAGGCGTGCAGGCTGAGCAAAGCGTAGGGA
TCATGGCGCAAAGATCGTTAGAGATGGCGATCGGAATCATCGCTATTCTCAAAGCGGGCGGGGCGTATGTGCC
GATCGATCCGGATTATCCGAATGAGCGGATTGCTTACATGCTGGAAGATTGCGAAGCCCGTCTGGTGCTGACC
CAGCAGCAGCTCGCCGAAAAGATGACCGCAAACGTGGAATGCCTGTATCTGGATGAGGAGGGCAGCTACTCG
CCTCAGACGGAAAACATCGAGCCGATCCATACCGCTGCTGATCTCGCTTACATCATCTACACATCCGGTACGA
CAGGCAGGCCAAAAGGCGTCATGGTAGAGCATCGGGGAATCGTCAACAGTGTGACGTGGAACAGGGACGAGT
TTGCCCTTTCTGTCCGGGACAGTGGAACGCTGTCGCTATCTTTTGCCTTCGATGCCTTTGCCCTTACTTTCTTTA
CGTTGATTGTATCAGGCTCCACGGTCGTCCTGATGCCGGATCACGAAGCCAAAGATCCGATCGCGCTACGCAA
CCTGATTGCCGCTTGGGAATGCAGCTACGTCGTTTTCGTGCCCAGTATGTTCCAGGCGATATTGGAGTGCAGCA
CTCCGGCAGACATCCGCTCCATCCAGGCAGTCGCGCTCGGGGGCGAAAAGCTGTCGCCGAAGCTTGTTCAGCT
GTGCAAAGCGATGCATCCGCAGATGAGCGTGATGAATGCATACGGCCCGACGGAGAGCAGCGTCAT**T**GCCAC
CTACCTGCGAGATACACAGCCAGATCAGCCGATCACCATCGGGCGGCCGATTGCCAACACCGCCATTTACATC
GTAGACCAGCACCATCAACTGCTGCCTGTCGGGGTGGTAGGGGAAATCTGCATCGGCGGTCACGGCTTGGCGC
GGGGCTATTGGAAAAAGCCGGAGCTTACTGCGGAGAAATTCGTGGCCAATCCAGCTGTTCCGGGAGAGCGCA
TGTACAAAACAGGCGATCTGGGCAGATGGCTCCACGACGGCACGATTGATTTTATAGGCCGCGTCGATGACCA
AATCAAGGTGAGAGGATACCGGATTGAGGTCGGGGAGATTGAAGCGGTTTTGCTCGCTTACGATCAGACGAA
TGAAGCTATCGTCGTCGCTTATCAGGACGATCGCGGCGATTCCTATCTGGCTGCGTATGTCACGGAAAAACG
GCGATAGAGGAATCCGAGCTTCGCGCGCATCTGTTGCGAGAGCTTCCGGCCTACATGGTGCCGACCTACCTGA
TTCAACTGGACGCTTTCCCGCTCACGCCAAACGGCAAGGTCGACCGCAAGGCACTGCCCAAGCCGGAAGGAA
AGCCTGCAACAGGAGCAGCTTATGTCGCACCCGCTACAGAAGTGGAGGCGAAGCTGGTCGCCATTTGGGAGA
ATGCGCTGGGGATTTCCGGCGTCGGGGTGTTGGATCACTTTTTTGAGCTGGGCGGTCATTCCTTGAAAGCGATG
ACGGTTGTGGCGCAAGTGCATCGCGAGTTTCAAATCGACCTTTTGCTGAAGCAGTTTTTTGCAGCGCCAACCAT
CCGGGACTTGGCCCGCTTGATCGAACATAGCGAACAGGCAGCCGGCGCCGCCATTCTCGAG**GAACAAAAGCT**
TATTTCTGAAGAGGACTTG

<u>pTrc99a-M1768A/M1803I-TycB-His</u> (The TycB start codon is in bold)

CTAGAGTCGACCTGCAGGCATGCAAGCTTGGCTGTTTTGGCGGATGAGAGAAGATTTTCAGCCTGATACAGAT
TAAATCAGAACGCAGAAGCGGTCTGATAAAACAGAATTTGCCTGGCGGCAGTAGCGCGGTGGTCCCACCTGA
CCCCATGCCGAACTCAGAAGTGAAACGCCGTAGCGCCGATGGTAGTGTGGGGTCTCCCCATGCGAGAGTAGG
GAACTGCCAGGCATCAAATAAAACGAAAGGCTCAGTCGAAAGACTGGGCCTTTCGTTTTATCTGTTGTTTGTC
GGTGAACGCTCTCCTGAGTAGGACAAATCCGCCGGGAGCGGATTTGAACGTTGCGAAGCAACGGCCCGGAGG
GTGGCGGGCAGGACGCCCGCCATAAACTGCCAGGCATCAAATTAAGCAGAAGGCCATCCTGACGGATGGCCT
TTTTGCGTTTCTACAAACTCTTTTGTTTATTTTTCTAAATACATTCAAATATGTATCCGCTCATGAGACAATAAC
CCTGATAAATGCTTCAATAATATTGAAAAAGGAAGAGTATGAGTATTCAACATTTCCGTGTCGCCCTTATTCCC
TTTTTTGCGGCATTTTGCCTTCCTGTTTTTGCTCACCCAGAAACGCTGGTGAAAGTAAAAGATGCTGAAGATCA
GTTGGGTGCACGAGTGGGTTACATCGAACTGGATCTCAACAGCGGTAAGATCCTTGAGAGTTTTCGCCCCGAA
GAACGTTTTCCAATGATGAGCACTTTTAAAGTTCTGCTATGTGGCGCGGTATTATCCCGTGTTGACGCCGGGCA
AGAGCAACTCGGTCGCCGCATACACTATTCTCAGAATGACTTGGTTGAGTACTCACCAGTCACAGAAAAGCAT
CTTACGGATGGCATGACAGTAAGAGAATTATGCAGTGCTGCCATAACCATGAGTGATAACACTGCGGCCAACT
TACTTCTGACAACGATCGGAGGACCGAAGGAGCTAACCGCTTTTTTGCACAACATGGGGGATCATGTAACTCG
CCTTGATCGTTGGGAACCGGAGCTGAATGAAGCCATACCAAACGACGAGCGTGACACCACGATGCCTACAGC
AATGGCAACAACGTTGCGCAAACTATTAACTGGCGAACTACTTACTCTAGCTTCCCGGCAACAATTAATAGAC
TGGATGGAGGCGGATAAAGTTGCAGGACCACTTCTGCGCTCGGCCCTTCCGGCTGGCTGGTTTATTGCTGATA
AATCTGGAGCCGGTGAGCGTGGGTCTCGCGGTATCATTGCAGCACTGGGGCCAGATGGTAAGCCCTCCCGTAT
CGTAGTTATCTACACGACGGGGAGTCAGGCAACTATGGATGAACGAAATAGACAGATCGCTGAGATAGGTGC
CTCACTGATTAAGCATTGGTAACTGTCAGACCAAGTTTACTCATATATACTTTAGATTGATTTAAAACTTCATT
TTTAATTTAAAAGGATCTAGGTGAAGATCCTTTTTGATAATCTCATGACCAAAATCCCTTAACGTGAGTTTTCG
TTCCACTGAGCGTCAGACCCCGTAGAAAAGATCAAAGGATCTTCTTGAGATCCTTTTTTTCTGCGCGTAATCTG
CTGCTTGCAAACAAAAAAACCACCGCTACCAGCGGTGGTTTGTTTGCCGGATCAAGAGCTACCAACTCTTTTT
CCGAAGGTAACTGGCTTCAGCAGAGCGCAGATACCAAATACTGTCCTTCTAGTGTAGCCGTAGTTAGGCCACC
ACTTCAAGAACTCTGTAGCACCGCCTACATACCTCGCTCTGCTAATCCTGTTACCAGTGGCTGCTGCCAGTGGC
GATAAGTCGTGTCTTACCGGGTTGGACTCAAGACGATAGTTACCGGATAAGGCGCAGCGGTCGGGCTGAACG
GGGGGTTCGTGCACACAGCCCAGCTTGGAGCGAACGACCTACACCGAACTGAGATACCTACAGCGTGAGCTA
TGAGAAAGCGCCACGCTTCCCGAAGGGAGAAAGGCGGACAGGTATCCGGTAAGCGGCAGGGTCGGAACAGG
AGAGCGCACGAGGGAGCTTCCAGGGGGAAACGCCTGGTATCTTTATAGTCCTGTCGGGTTTCGCCACCTCTGA
CTTGAGCGTCGATTTTTGTGATGCTCGTCAGGGGGGCGGAGCCTATGGAAAAACGCCAGCAACGCGGCCTTTT
TACGGTTCCTGGCCTTTTGCTGGCCTTTTGCTCACATGTTCTTTCCTGCGTTATCCCCTGATTCTGTGGATAACC
GTATTACCGCCTTTGAGTGAGCTGATACCGCTCGCCGCAGCCGAACGACCGAGCGCAGCGAGTCAGTGAGCG
AGGAAGCGGAAGAGCGCCTGATGCGGTATTTTCTCCTTACGCATCTGTGCGGTATTTCACACCGCATATGGTG
CACTCTCAGTACAATCTGCTCTGATGCCGCATAGTTAAGCCAGTATACACTCCGCTATCGCTACGTGACTGGGT
CATGGCTGCGCCCCGACACCCGCCAACACCCGCTGACGCGCCCTGACGGGCTTGTCTGCTCCCGGCATCCGCT
TACAGACAAGCTGTGACCGTCTCCGGGAGCTGCATGTGTCAGAGGTTTTCACCGTCATCACCGAAACGCGCGA
GGCAGCAGATCAATTCGCGCGCGAAGGCGAAGCGGCATGCATTTACGTTGACACCATCGAATGGTGCAAAAC
CTTTCGCGGTATGGCATGATAGCGCCCGGAAGAGAGTCAATTCAGGGTGGTGAATGTGAAACCAGTAACGTTA
TACGATGTCGCAGAGTATGCCGGTGTCTCTTATCAGACCGTTTCCCGCGTGGTGAACCAGGCCAGCCACGTTT
CTGCGAAAACGCGGGAAAAAGTGGAAGCGGCGATGGCGGAGCTGAATTACATTCCCAACCGCGTGGCACAAC
AACTGGCGGGCAAACAGTCGTTGCTGATTGGCGTTGCCACCTCCAGTCTGGCCCTGCACGCGCCGTCGCAAAT
TGTCGCGGCGATTAAATCTCGCGCCGATCAACTGGGTGCCAGCGTGGTGGTGTCGATGGTAGAACGAAGCGGC
GTCGAAGCCTGTAAAGCGGCGGTGCACAATCTTCTCGCGCAACGCGTCAGTGGGCTGATCATTAACTATCCGC
TGGATGACCAGGATGCCATTGCTGTGGAAGCTGCCTGCACTAATGTTCCGGCGTTATTTCTTGATGTCTCTGAC
CAGACACCCATCAACAGTATTATTTTCTCCCATGAAGACGGTACGCGACTGGGCGTGGAGCATCTGGTCGCAT
TGGGTCACCAGCAAATCGCGCTGTTAGCGGGCCCATTAAGTTCTGTCTCGGCGCGTCTGCGTCTGGCTGGCTG
GCATAAATATCTCACTCGCAATCAAATTCAGCCGATAGCGGAACGGGAAGGCGACTGGAGTGCCATGTCCGG
TTTTCAACAAACCATGCAAATGCTGAATGAGGGCATCGTTCCCACTGCGATGCTGGTTGCCAACGATCAGATG
GCGCTGGGCGCAATGCGCGCCATTACCGAGTCCGGGCTGCGCGTTGGTGCGGATATCTCGGTAGTGGGATACG
ACGATACCGAAGACAGCTCATGTTATATCCCGCCGTTAACCACCATCAAACAGGATTTTCGCCTGCTGGGGCA
AACCAGCGTGGACCGCTTGCTGCAACTCTCTCAGGGCCAGGCGGTGAAGGGCAATCAGCTGTTGCCCGTCTCA
CTGGTGAAAAGAAAAACCACCCTGGCGCCCAATACGCAAACCGCCTCTCCCCGCGCGTTGGCCGATTCATTAA
TGCAGCTGGCACGACAGGTTTCCCGACTGGAAAGCGGGCAGTGAGCGCAACGCAATTAATGTAAGTTAGCGC
GAATTGATCTGGTTTGACAGCTTATCATCGACTGCACGGTGCACCAATGCTTCTGGCGTCAGGCAGCCATCGG
AAGCTGTGGTATGGCTGTGCAGGTCGTAAATCACTGCATAATTCGTGTCGCTCAAGGCGCACTCCCGTTCTGG
ATAATGTTTTTTGCGCCGACATCATAACGGTTCTGGCAAATATTCTGAAATGAGCTGTTGACAATTAATCATCC
GGCTCGTATAATGTGTGGAATTGTGAGCGGATAACAATTTCACACAGGAAAGGTACC**ATG**AGTGTATTTAGCA

AAGAACAAGTTCAGGATATGTATGCGTTGACCCCGATGCAAGAGGGGATGCTGTTTCACGCCTTGCTCGATCA
AGAGCACAACTCGCATCTGGTACAGATGTCGATTTCGTTGCAGGGCGATCTTGACGTTGGGCTATTTACGGAT
AGCCTGCATGTGCTGGTAGAGAGATACGATGTATTCCGCACGTTGTTTCTCTATGAAAAGCTGAAGCAGCCTT
TGCAAGTTGTCTTGAAGCAACGGCCTATTCCGATCGAATTTTACGACTTGTCTGCCTGCGACGAGTCCGAGAA
ACAACTTCGCTATACGCAATACAAAAGGGCGGATCAGGAGCGGACGTTTCATCTGGCAAAAGACCCGTTGAT
GCGGGTCGCCCTTTTCCAAATGTCCCAGCACGACTACCAGGTCATCTGGAGCTTTCATCACATCCTCATGGACG
GCTGGTGCTTCAGCATTATTTTTGATGACTTGCTTGCCATCTACTTGTCCTTGCAAAACAAGACGGCACTCTCC
CTGGAGCCCGTACAGCCATACAGTCGCTTTATCAACTGGCTGGAAAAACAAAATAAACAGGCCGCTCTCAACT
ATTGGAGCGACTATCTGGAAGCCTATGAACAAAAGACTACCTTGCCGAAGAAGGAAGCTGCCTTCGCCAAAG
CATTTCAACCAACCCAATACCGCTTTTCGCTGAACCGCACCTTGACCAAGCAGCTCGGGACCATCGCCAGTCA
AAATCAAGTGACGCTATCGACGGTGATTCAAACGATCTGGGGAGTTCTCCTGCAAAAATACAATGCGGCCCAT
GATGTGCTGTTCGGCTCTGTTGTATCCGGACGCCCTACAGACATCGTCGGAATCGACAAAATGGTTGGCTTGTT
TATCAATACGATTCCATTCCGGGTGCAAGCGAAAGCTGGTCAAACGTTTTCCGAGCTGTTGCAAGCTGTGCAC
AAAAGAACTTTGCAATCACAGCCGTATGAGCACGTGCCTTTGTACGACATTCAAACTCAGTCCGTCTTGAAGC
AGGAGCTGATTGACCACCTGCTGGTCATCGAAAATTACCCGCTGGTAGAGGCTTTGCAGAAAAAAGCATTGAA
CCAGCAGATCGGCTTCACGATTACTGCTGTGGAAATGTTCGAGCCGACCAATTACGACTTGACTGTCATGGTG
ATGCCAAAAGAAGAGCTTGCCTTCCGTTTTGACTACAATGCGGCTCTGTTTGACGAACAGGTCGTGCAAAAAC
TGGCGGGGCACCTCCAACAGATCGCGGATTGCGTGGCAAACAATTCGGGAGTCGAGCTTTGCCAGATTCCGTT
GCTGACAGAAGCAGAAACTAGCCAGCTGTTGGCAAAGCGTACGGAAACAGCGGCTGACTATCCTGCCGCAAC
CATGCACGAGCTGTTTTCGCGGCAGGCAGAAAAAACGCCTGAGCAAGTGGCGGTAGTCTTCGCGGATCAGCA
CCTGACGTATCGGGAGCTGGATGAAAAATCCAATCAGCTCGCCCGCTTTTTGCGCAAAAAAGGCATTGGCACG
GGCAGTCTTGTCGGCACGCTGCTGGATCGCTCGCTGGACATGATCGTCGGAATCCTCGGCGTCTTGAAGGCAG
GCGGCGCATTTGTGCCGATCGACCCGGAGTTGCCTGCCGAACGAATCGCTTACATGCTGACGCATAGCAGAGT
TCCATTGGTCGTGACGCAAAATCATTTGCGGGCAAAAGTGACCACGCCTACAGAAACAATTGACATCAACAC
AGCGGTGATCGGGGAAGAGAGCCGCGCCCCTATCGAATCGCTCAATCAGCCGCATGACTTGTTTTACATCATC
TATACGTCCGGAACGACAGGGCAACCGAAAGGCGTCATGCTGGAGCATCGCAACATGGCGAACCTGATGCAT
TTTACGTTTGATCAGACGAACATCGCTTTTCATGAAAAAGTGTTGCAGTATACCACGTGCAGCTTTGATGTTTG
CTACCAGGAAATTTTCTCCACGCTGCTATCCGGGGGCCAGCTCTACCTGATCACGAACGAGCTGAGACGGCAT
GTGGAAAAGCTGTTTGCTTTCATCCAGGAAAAGCAGATCAGCATTTTGTCTCTCCCGGTGTCCTTCCTGAAATT
TATTTTTAACGAACAAGACTACGCGCAAAGCTTCCCGCGTTGTGTCAAACATATCATCACGGCCGGGGAACAA
CTCGTCGTCACACACGAGCTGCAAAAGTATCTGCGCCAGCATCGCGTATTTTTGCACAATCACTACGGCCCGT
CGGAGACGCATGTGGTGACGACATGCACGATGGACCCGGGACAGGCGATACCAGAGCTGCCGCCCATCGGAA
AGCCGATCAGCAACACAGGCATTTACATTTTGGATGAAGGGCTGCAATTGAAGCCGGAGGGGATCGTCGGGG
AGTTGTACATTTCCGGCGCAAACGTAGGAAGAGGGTATTTGCACCAGCCGGAGCTGACCGCGGAGAAGTTTCT
CGACAATCCGTATCAGCCAGGCGAAAGAATGTACCGAACGGGTGATCTGGCGCGTTGGTTGCCGGATGGCCA
GCTCGAATTTTTGGGCCGAATCGACCATCAGGTAAAAATCAGGGGCCATCGCATCGAGCTGGGAGAGATCGA
ATCGCGCCTGCTCAACCATCCCGCCATCAAGGAAGCGGTGGTTATCGACCGAGCAGACGAGACAGGCGGCAA
GTTTTTGTGCGCCTATGTCGTCCTGCAAAAAGCGCTCAGCGACGAAGAGATGCGGGCATACTTGGCGCAAGCG
TTGCCGGAGTATATGATCCCTTCCTTTTTCGTGACGCTGGAGCGGATTCCAGTCACGCCGAACGGAAAAACAG
ACAGGCGAGCTTTGCCGAAGCCGGAAGGAAGTGCCAAGACGAAAGCGGATTACGTCGCCCCGACGACTGAGC
TGGAACAAAAGCTGGTCGCGATTTGGGAGCAAATTCTTGGCGTGTCGCCGATCGGCATTCAGGATCATTTTTT
CACGCTGGGCGGCCATTCGTTAAAAGCGATTCAGCTCATTTCCCGCATCCAAAAGGAATGCCAGGCGGATGTC
CCGCTGCGCGTCCTGTTTGAGCAACCGACGATTCAAGCGCTGGCAGCGTATGTGGAAGGCGGGGAGGAAAGC
GCGTATCTCGCCATTCCCCAGGCCGAGCCGCAAGCGTATTATCCCGTATCGTCTGCGCAAAAACGCATGCTCA
TCTTAAACCAGCTCGATCCGCACAGCACGGTTTACAACCTGCCTGTCGCGATGATCCTCGAAGGAACGCTGGA
TAAAGCTCGGCTGGAGCACGCCATTTCCAACCTGGTGGCTCGCCATGAGTCGTTGCGCACGTCGTTTCATACG
ATCAACGGGGAGCCAGTTTCCCGCATCCATGAGCAAGGCCACTTGCCGATTGTTTACTTGGAAACGGCGGAAG
AGCAAGTGAACGAGGTCATTTTGGGGTTCATGCAGCCGTTTGATCTGGTAACAGCTCCGCTATGCCGGGTTGG
CTTGGTGAAGCTCGCAGAGAACCGTCACGTCCTGATCATCGACATGCACCATATCATTTCGGACGGAGTCTCT
TCTCAGCTCATCCTGAATGACTTTTCCCGTTTGTATCAAAACAAAGCTTTGCCAGAGCAGCGCATTCACTATAA
AGACTTCGCCGTTTGGGAAAAAGCGTGGACACAAACGACCGATTACCAAAAACAGGAAAAATATTGGCTCGA
TCGATTTGCGGGCGAAATCCCGGTTTTGAACCTGCCGATGGATTACCCGCGGCCAGCTGTTCAAAGCTTTGAG
GGCGAACGTTATTTGTTCCGCACAGAAAAACAGTTGTTGGAAAGTTTGCAGGACGTAGCCCAAAAGACAGGC
ACGACCTTGTACATGGTGCTTCTCGCAGCCTATCATGTGCTGCTTTCCAAATACTCCGGGCAGGATGACGTGAT
GATCGGCACCGTGACTGCCGGCAGGGTGCACCCGGATACGGAGAGCATGACGGGGATGTTCGTCAACACGCT
GGCGATGCGCAATCAGTCTGCGCCGACCAAAACGTTCCGGCAATTTTTGCTGGAGGTAAAAGACAACACGCT
GGCCGCTTTTGAACACGGGCAATATCCGTTTGAAGAGCTTGTCGAAAAGTTGGCGATCCAGCGAAACCGGAGC
CGAAACCCGCTGTTCGACACCTTGTTCATTTTGCAAAACATGGATGCCGACCTGATCGAGCTGGATGGACTGA

CCGTGACGCCTTATGTGCCAGAGGGGGAAGTCGCCAAGTTCGATCTGTCGCTGGAAGCAAGCGAAAACCAGG
CGGGACTTTCCTTCTGCTTCGAATTTTGCACCAAGCTGTTCGCACGCGAGACGATCGAGCGCATGTCGCTTCAT
TACTTGCAAATTTTGCAGGCAGTCAGCGCAAACACGGAGCAGGAGCTGGCGCAAATCGAGATGCTGACTGCG
CATGAGAAGCAGGAGCTGCTCGTTCACTTCAACGACACGGCCGCCCTGTATCCAGCGGAGAGCACGCTGTCGC
AGCTGTTTGAAGATCAGGCACAGAAAACTCCTGAGCAAACCGCCGTCGTCTTCGGTGACAAACGACTGACGT
ACCGCGAACTGAACGAGCGGGCCAACCAGCTCGCGCACACTTTGCGGGCAAAAGGCGTGCAGGCTGAGCAAA
GCGTAGGGATCATGGCGCAAAGATCGCTCGAGATGGCGATCGGAATCATCGCTATTCTCAAAGCGGGCGGGG
CGTATGTGCCGATCGATCCGGATTATCCGAATGAGCGGATTGCTTACATGCTGGAAGATTGCGAAGCCCGTCT
GGTGCTGACCCAGCAGCAGCTCGCCGAAAAGATGACCGCAAACGTGGAATGCCTGTATCTGGATGAGGAGGG
CAGCTACTCGCCTCAGACGGAAAACATCGAGCCGATCCATACCGCTGCTGATCTCGCTTACATCATCTACACA
TCCGGTACGACAGGCAGGCCAAAAGGCGTCATGGTAGAGCATCGGGGAATCGTCAACAGTGTGACGTGGAAC
AGGGACGAGTTTGCCCTTTCTGTCCGGGACAGTGGAACGCTGTCGCTATCTTTTGCCTTCGATGCCTTTGCCCT
TACTTTCTTTACGTTGATTGTATCAGGCTCCACGGTCGTCCTGATGCCGGATCACGAAGCCAAAGATCCGATCG
CGCTACGCAACCTGATTGCCGCTTGGGAATGCAGCTACGTCGTTTTCGTGCCCAGTATGTTCCAGGCGATATTG
GAGTGCAGCACTCCGGCAGACATCCGCTCCATCCAGGCAGTC**GC**GCTCGGGGGCGAAAAGCTGTCGCCGAAG
CTTGTTCAGCTGTGCAAAGCGATGCATCCGCAGATGAGCGTGATGAATGCATACGGCCCGACGGAGAGCAGC
GTCAT**T**GCCACCTACCTGCGAGATACACAGCCAGATCAGCCGATCACCATCGGGCGGCCGATTGCCAACACCG
CCATTTACATCGTAGACCAGCACCATCAACTGCTGCCTGTCGGGGTGGTAGGGGAAATCTGCATCGGCGGTCA
CGGCTTGGCGCGGGGCTATTGGAAAAAGCCGGAGCTTACTGCGGAGAAATTCGTGGCCAATCCAGCTGTTCCG
GGAGAGCGCATGTACAAAACAGGCGATCTGGGCAGATGGCTCCACGACGGCACGATTGATTTTATAGGCCGC
GTCGATGACCAAATCAAGGTGAGAGGATACCGGATTGAGGTCGGGGAGATTGAAGCGGTTTTGCTCGCTTAC
GATCAGACGAATGAAGCTATCGTCGTCGCTTATCAGGACGATCGCGGCGATTCCTATCTGGCTGCGTATGTCA
CGGGAAAAACGGCGATAGAGGAATCCGAGCTTCGCGCGCATCTGTTGCGAGAGCTTCCGGCCTACATGGTGC
CGACCTACCTGATTCAACTGGACGCTTTCCCGCTCACGCCAAACGGCAAGGTCGACCGCAAGGCACTGCCCAA
GCCGGAAGGAAAGCCTGCAACAGGAGCAGCTTATGTCGCACCCGCTACAGAAGTGGAGGCGAAGCTGGTCGC
CATTTGGGAGAATGCGCTGGGGATTTCCGGCGTCGGGGTGTTGGATCACTTTTTTGAGCTGGGCGGTCATTCCT
TGAAAGCGATGACGGTTGTGGCGCAAGTGCATCGCGAGTTTCAAATCGACCTTTTGCTGAAGCAGTTTTTTGC
AGCGCCAACCATCCGGGACTTGGCCCGCTTGATCGAACATAGCGAACAGGCAGCCGGCGCCGCCATTCAACC
GGCAGAGCCGCAAGCGTATTATCCGGTATCTTCTGCTCAGCAGCGGATGTACTTGCTCCATCAGCTTGAAGGT
GCCGGAATCAGCTACAACACACCGGGCATCATCATGCTGGAAGGCAAGCTCGATCGCGAGCAATTGGCGAAT
GCGCTGCAAGCGTTGGTAGATCGTCACGATATTTTGCGGACGTCTTTTGAGATGGTCGGAGACGAGCTGGTGC
AAAAAATTCATGACCGCGTGGCCGTGAACATGGAGTATGTGACGGCAGAAGAGCAGCAGATCGATGACCTTT
TCCACGCGTTCGTCCGTCCGTTTGATCTTTCTGTGCCGCCATTGCTCCGCATGAGCCTGGTGAAACTCGCGGAT
GAGCGTCACCTGCTCCTGTACGACATGCACCATATTGCTGCCGATGCCGCATCGATCACGATCCTGTTCGATG
AACTGGCTGAATTGTACCAGGGAAGAGAACTGCCGGAAATGCGCATCCAGTACAAAGATTTTGCTGTCTGGCA
AAAAGCCTTGCATGAGTCGGATGCCTTCAAGCAGCAGGAAGCGTATTGGCTGAGCACGTTCGCTGGAAATATC
ACCGCTGTCGATGTGCCGACAGATTTTCCGCGCCCAGCCGTGAAAAGTTTTGCAGGGGGGCAAGTCACCCTGT
CCATGGACCAAGAGCTGCTCAGTGCTTTGCACGAGTTGGCTGCGCATACGAATACGACGCTGTTTATGGTTTT
GCTGGCCGCCTACAACGTGCTGCTCGCAAAATACGCTGGGCAAGACGACATCATCGTGGGAACGCCGATCTCC
GGCAGGTCACGCGCCGAGCTTGCGCCTGTCGTCGGCATGTTCGTCCATACGCTGGCGATCCGCAACAAACCGA
CCGCCGAGAAGACATTCAAGCAGTTTTTGCAGGAGGTCAAGCAAAACGCGCTCGATGCTTTCGACCACCAGG
ACTACCCGTTTGAAAGCCTTGTGGAAAAGCTGGGCATTCCGCGCGATCCGGGGCGCAATCCGCTGTTTGACAC
CATGTTCATCCTGCAAAACGATGAGTTGCACGCAAAAACGCTGGATCAGCTCGTCTATCGCCCTTATGAATCG
GACAGCGCGCTTGACGTGGCGAAATTCGACTTGTCGTTCCATCTGACCGAGCGGGAAACCGACCTGTTCTTGC
GCCTGGAATACTGCACCAAGCTGTTCAAGCAACAAACGGTAGAACGAATGGCGCACCACTTCTTGCAAATTTT
GCGAGCGGTCACGGCCAATCCGGAAAATGAATTGCAAGAGATCGAGATGCTGACAGCAGCAGAAAAGCAAA
TGCTGCTGGTGGCGTTCAACGATACGCACAGAGAATACCGGGCAGATCAAACAATCCAGCAACTTTTTGAAGA
GCTGGCGGAAAAAATGCCTGAGCACACGGCGCTCGTATTCGAAGAAAAGCGCATGTCGTTCCGGGAGCTCAA
TGAAAGAGCGAACCAGCTCGCAGCCGTTTTGCGGGAAAAAGGAGTCGGGCCAGCGCAGATCGTCGCTTTGCT
GGTAGAGCGTTCCGCCGAGATGGTCATTGCCACGCTTGCCACGTTAAAAGCGGGCGGCGCCTTTTTGCCCGTC
GATCCTGATTATCCGGAAGAGCGAATCCGCTACATGCTGGAGGACAGCCAGGCAAAACTGGTGGTGACCCAT
GCGCACTTGCTGCACAAAGTGAGCAGTCAGTCCGAAGTCGTTGATGTGGATGACCCTGGAAGCTACGCAACA
CAGACAGACAACCTGCCGTGCGCAAACACACCGTCTGATTTGGCTTATATCATTTACACGTCCGGTACGACGG
GCAAGCCAAAAGGCGTCATGCTGGAGCACAAAGGGGTAGCGAATCTGCAAGCGGTATTTGCCCATCATCTAG
GCGTCACGCCGCAAGATCGGGCAGGGCATTTTGCCAGCATCTCGTTTGACGCATCGGTGTGGGATATGTTTGG
CCCGTTGCTGTCGGGAGCGACCTTGTACGTCTTGTCCCGAGACGTCATCAACGATTTTCAACGATTCGCCGAAT
ACGTTCGCGATAACGCGATCACCTTCCTCACTTTGCCGCCGACGTACGCGATTTATCTGGAGCCGGAGCAGGT
GCCGTCGTTACGCACCCTGATTACAGCCGGATCGGCTTCCTCCGTTGCATTGGTGGATAAATGGAAAGAAAAA

GTCACCTATGTCAATGGATACGGCCCAACAGAGAGCACCGTTTGCGCGACACTGTGGAAAGCCAAACCGGAT
GAGCCAGTCGAAACGATCACGATTGGCAAACCGATTCAGAACACCAAGCTGTACATCGTGGATGACCAGTTG
CAGTTGAAAGCGCCGGGGCAGATGGGAGAACTGTGCATCAGCGGCTTGTCGCTGGCGAGAGGCTATTGGAAT
CGTCCAGAGCTGACCGCCGAGAAGTTCGTCGACAACCCGTTTGTGCCAGGAACAAAGATGTACCGGACAGGC
GACCTGGCAAGATGGCTGCCAGATGGAACTATCGAGTATCTGGGCAGAATCGATCACCAAGTGAAAATTCGC
GGACATCGTGTGGAACTCGGCGAAGTGGAAAGCGTGCTGCTGCGGTATGACACGGTCAAAGAGGCGGCCGCC
ATCACACATGAGGACGACCGCGGCCAAGCTTACTTGTGCGCCTACTACGTAGCGGAGGGAGAAGCCACGCCT
GCGCAACTTCGAGCCTATATGGAAAACGAGTTGCCGAACTACATGGTTCCCGCCTTCTTTATCCAGTTGGAAA
AGATGCCGCTGACACCGAATGACAAGATTGACCGAAAAGCGCTGCCGAAGCCGAACCAGGAGGAGAACCGG
ACTGAGCAATATGCAGCGCCGCAAACCGAGCTGGAACAGTTGCTGGCTGGCATCTGGGCAGATGTACTGGGG
ATCAAGCAAGTCGGGACGCAAGACAACTTCTTTGAATTGGGCGGCGATTCGATTAAAGCGATCCAGGTATCCA
CCCGCCTGAATGCGTCAGGCTGGACGCTTGCGATGAAAGAACTGTTCCAGTACCCGACGATTGAAGAAGCTGC
TCTGCGCGTCATCCCGAACAGCCGAGAGAGCGAGCAGGGTGTCGTAGAAGGCGAGATTGCCTTGACACCGAT
CCAGAAATGGTTCTTCGCGAACAACTTCACGGATCGTCACCATTGGAATCAGGCTGTCATGCTGTTTCGCGAG
GACGGCTTTGACGAGGGACTCGTGCGGCAAGCGTTCCAGCAAATCGTCGAGCATCACGATGCGCTGCGCATG
GTCTACAAGCAAGAGGACGGGGCGATCAAGCAAATCAACCGCGGGCTGACCGACGAGCGCTTCCGTTTCTAC
TCTTATGACTTGAAAAATCACGCGAACAGCGAAGCTCGCATCCTGGAGCTGTCTGATCAGATCCAGAGCAGCA
TCGATTTGGAGCACGGCCCACTCGTTCACGTGGCTCTGTTCGCCACAAAAGACGGGGATCATTTGCTGGTCGC
GATCCACCATCTTGTCGTGGATGGCGTCTCCTGGCGCATTTTGTTCGAAGATTTTTCCTCAGCCTACTCGCAGG
CTCTCCATCAGCAGGAGATCGTCTTGCCGAAAAAGACGGACTCCTTCAAAGACTGGGCGGCTCAATTGCAAAA
GTACGCGGACAGCGACGAGCTGTTGCGGGAAGTGGCATATTGGCACAACTTGGAGACTACAACGACGACTGC
GGCACTGCCAACAGATTTTGTCACGGCAGATCGCAAGCAAAAACATACGCGGACACTGTCGTTCGCGTTGACA
GTCCCGCAGACAGAAAACCTTTTGCGTCACGTTCATCATGCCTATCACACAGAGATGAACGACCTGCTGCTGA
CAGCGCTCGGCTTGGCCGTAAAAGACTGGGCACATACGAATGGCGTCGTCATCAATCTGGAAGGCCATGGGC
GCGAAGACATCCAGAACGAAATGAACGTCACGCGCACGATTGGCTGGTTCACTTCGCAATATCCGGTGGTGCT
CGACATGGAAAAAGCCGAGGACTTGCCGTACCAGATCAAGCAAACCAAAGAAAACTTGCGACGGATTCCGAA
AAAAGGGATCGGCTACGAGATTTTGCGCACGCTGACGACAAGCCAGTTGCAGCCGCCATTAGCCTTTACGCTG
CGGCCGGAAATCAGCTTTAACTATCTCGGTCAATTCGAGTCGGACGGAAAAACAGGCGGGTTTACATTCTCGC
CGCTCGGAACAGGGCAGTTGTTCAGCCCGGAATCGGAGCGAGTGTTCCTGCTGGACATTTCCGCCATGATCGA
GGACGGCGAGCTGCGGATCAGCGTGGGGTACAGCCGTCTCCAATATGAGGAAAAAACGATTGCCAGCCTGGC
AGACAGCTACCGGAAGCACTTGCTAGGCATCATCGAGCATTGCATGGCAAAAGAAGAAGGCGAGTACACCCC
GAGCGACCTGGGGGATGAAGAGCTGTCCATGGAGGAGCTGGAAAACATCCTGGAATGGATTGGATCCAGATC
TCATCACCATCACCATCACGCTTAAT

References

(1) Kries, H.; Wachtel, R.; Pabst, A.; Wanner, B.; Niquille, D.; Hilvert, D. Reprogramming nonribosomal peptide synthetases for "clickable" amino acids. *Angew. Chem. Int. Ed.* **2014**, 53, 10105-10108.

(2) Gruenewald, S.; Mootz, H. D.; Stehmeier, P.; Stachelhaus, T. In vivo production of artificial nonribosomal peptide products in the heterologous host *Escherichia coli. Appl. Environ. Microbiol.* **2004**, *70*, 3282–3291.

(3) Niquille, D. L.; Hansen, D. A.; Mori, T.; Fercher, D.; Kries, H.; Hilvert, D. Nonribosomal biosynthesis of backbone-modified peptides. *Nat. Chem.* **2018**, *10*, 282–287.

(4) Niquille, D. L.; Folger, I. B.; Basler, S.; Hilvert, D. Biosynthetic functionalization of nonribosomal peptides. *J. Am. Chem. Soc.* **2021**, *143*, 2736–2740.

(5) Boder, E. T.; Wittrup, K. D. Yeast surface display for directed evolution of protein expression, affinity, and stability. *Methods Enzymol.* **2000**, *328*, 430–444.

(6) Blomberg, R. Optimizing artificial enzymes with directed evolution, ETH Zurich, 2011.

(7) Benatuil, L.; Perez, J. M.; Belk, J.; Hsieh, C. M. An improved yeast transformation method for the generation of very large human antibody libraries. *Protein Eng. Des. Sel.* **2010**, *23*, 155–159.

(8) Reuter, K. Crystal structure of the surfactin synthetase-activating enzyme Sfp: a prototype of the 4'-phosphopantetheinyl transferase superfamily. *EMBO J.* **1999**, *18*, 6823–6831.

(9) Lambalot, R. H.; Gehring, A. M.; Flugel, R. S.; Zuber, P.; LaCelle, M.; Marahiel, M. A.; Reid, R.; Khosla, C.; Walsh, C. T. A new enzyme superfamily - The phosphopantetheinyl transferases. *Chem. Biol.* **1996**, *3*, 923–936.

(10) Studier, F. W. Protein production by auto-induction in high density shaking cultures. *Protein Expr. Purif.* **2005**, *41*, 207–234.

(11) Tanovic, A.; Samel, S. A.; Essen, L.-O.; Marahiel, M. A. Crystal structure of the termination module of a nonribosomal peptide synthetase. *Science* **2008**, *321*, 659–663.

(12) Samel, S. A.; Schoenafinger, G.; Knappe, T. A.; Marahiel, M. A.; Essen, L. O. Structural and Functional Insights into a Peptide Bond-Forming Bidomain from a Nonribosomal Peptide Synthetase. *Structure* **2007**, *15* , 781–792.

(13) Stachelhaus, T.; Mootz, H. D.; Marahiel, M. A. The specificity-conferring code of adenylation domains in nonribosomal peptide synthetases. *Chem. Biol.* **1999**, *6*, 493–505.

(14) Rausch, C.; Weber, T.; Kohlbacher, O.; Wohlleben, W.; Huson, D. H. Specificity Prediction of Adenylation Domains in Nonribosomal Peptide Synthetases (NRPS) Using Transductive Support Vector Machines (TSVMs). *Nucleic Acids Res.* **2005**, *33*, 5799–5808.
